# Astaxanthin as a Novel Mitochondrial Regulator: A New Aspect of Carotenoids, beyond Antioxidants

**DOI:** 10.3390/nu14010107

**Published:** 2021-12-27

**Authors:** Yasuhiro Nishida, Allah Nawaz, Karen Hecht, Kazuyuki Tobe

**Affiliations:** 1 First Department of Internal Medicine, Faculty of Medicine, University of Toyama, 2630 Sugitani, Toyama 930-0194, Japan; 2 Fuji Chemical Industries, Co., Ltd., 55 Yokohoonji, Kamiich-machi, Nakaniikawa-gun, Toyama 930-0405, Japan; 3 Department of Molecular and Medical Pharmacology, Faculty of Medicine, University of Toyama, 2630 Sugitani, Toyama 930-0194, Japan; 4 AstaReal, Inc., 3 Terri Lane, Unit 12, Burlington, NJ 08016, USA; khecht@astarealusa.com

**Keywords:** astaxanthin, obesity, mitochondria, energy metabolisms, natural antioxidant, insulin resistance, AMPK

## Abstract

Astaxanthin is a member of the carotenoid family that is found abundantly in marine organisms, and has been gaining attention in recent years due to its varied biological/physiological activities. It has been reported that astaxanthin functions both as a pigment, and as an antioxidant with superior free radical quenching capacity. We recently reported that astaxanthin modulated mitochondrial functions by a novel mechanism independent of its antioxidant function. In this paper, we review astaxanthin’s well-known antioxidant activity, and expand on astaxanthin’s lesser-known molecular targets, and its role in mitochondrial energy metabolism.

## 1. Introduction

### 1.1. Hidden Bioactivity of Natural Pigments

#### 1.1.1. Nature Is Full of Splendid Color!

When we look at the natural world around us, we can find a biodiversity of colors in both plants and animals. Colors can be formed when light is absorbed and reflected by pigments and dyes, or when light scatters from micro- and nanostructures to form structural colors. In nature, most colors are produced by pigments derived from both organic and mineral sources. Major organic pigment types include the following: porphyrins, such as green chlorophylls and red hemes; flavonoids, such as blue-purple anthocyanins of flowers and fruits; and carotenoids, a large group of yellow, orange, and red pigments found in plants, algae, bacteria, and fungi [1]. In addition to contributing color, pigments also have a great variety of documented physiological activities [2,3,4,5].

In this review, the biological activities of carotenoids, especially those of astaxanthin (AX), are discussed. In particular, the interesting effects of AX on mitochondria in the context of physical performance, metabolic and aging disorders, and cancer, have been addressed. The antioxidant activity of AX is one of its most often cited mechanisms of action, but additional effects of AX on mitochondria have been observed that may not be directly related to its antioxidant activity. The focus of this review is to discuss existing evidence of AX’s additional biological activities, beyond its well-known antioxidant properties.

#### 1.1.2. Carotenoids

**Figure 1:**
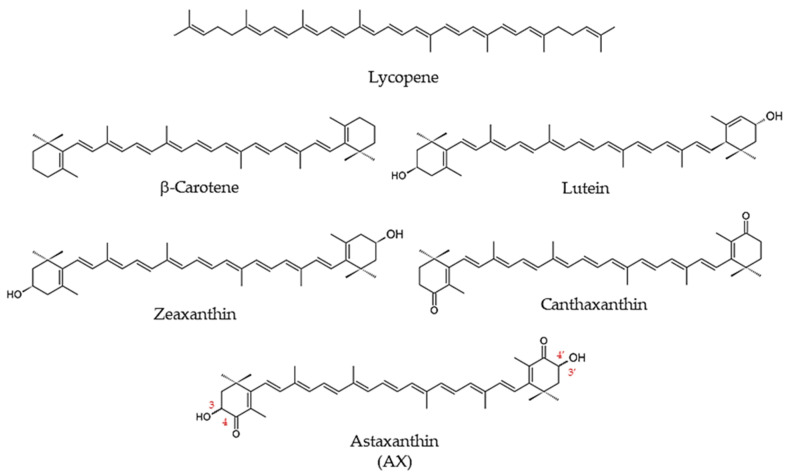
Structure of astaxanthin (AX) and related carotenoids.

Most carotenoids are strongly lipophilic, including β-carotene—found abundantly in carrots—and lycopene, which gives tomatoes and watermelons their red color [1] (Figure 1). In animals, many carotenoids, such as β-carotene, are known as provitamin A carotenoids, because they serve as precursors in the metabolic synthesis of vitamin A and its derivatives [1]. With few exceptions, such as some arthropods, animals cannot synthesize carotenoids de novo [6]. Therefore, animals depend on dietary sources for a supply of carotenoids.

#### 1.1.3. What Is Astaxanthin?

AX is a carotenoid that is frequently found in aquatic organisms, where it contributes its bright orange-to-red color, as in the shells of shrimp and crab, and in the muscles of salmon and trout [7,8,9]. Although AX is best recognized as a pigment characteristic of aquatic organisms, it’s extensive presence in prokaryotes and eukaryotes is less commonly known. AX is a derivative of β-carotene, bearing a similar structure that differs at its terminal β-ionone rings. In contrast to β-carotene, the β-ionone rings of AX have hydroxyl groups at the 3,3′-positions, and keto groups at the 4,4′-positions. The long central polyene chain consists of conjugated double bonds (Figure 1).

Unlike β-carotene, AX shows negligible pro-vitamin A activity, except under unusual conditions such as severe vitamin A deficiency [10]. The carbons attached to the hydroxyl groups at both ends are chiral, producing optical isomers that differ based on the orientations of the hydroxyl groups. The hydroxyl groups of AX can bind to fatty acids, sugars, or proteins. In addition, the central polyene chain often has an all-trans configuration, but there are also geometric isomers, in which portion(s) of AX may bear a *cis* configuration [9].

Although AX has been observed in an abundant number of aquatic organisms, its concentration greatly differs across species and tissues [7,8,11,12]. To mimic its presence in wild animals, AX is utilized in animal feed to improve the color of fish and poultry. Most of the AX in circulation for aquaculture and feed is chemically synthesized, with a smaller proportion being derived from natural sources, such as basidiomycete yeast, called *Phaffia* (official name: *Xanthophyllomyces dendrorhous*); flower petals, derived from *Adonis aestivalis*; and bacteria, derived from *Paracoccus* sp.

Based on its long-standing presence in the human diet, and an abundant number of published safety studies, AX is considered safe for food consumption, and has been used as a functional food additive for humans in recent years. The most common source of AX used in functional foods and supplements comes from a unicellular green alga called *Haematococcus*, with krill representing a more minor secondary source.

*Haematococcus* algae are green and motile cells during their active growing or vegetative state, until the growth environment becomes unfavorable due to nutrient starvation, high light conditions, or high osmotic pressure. In response to such adverse growth conditions, the algal cells transition into a resting state in which they accumulate high concentrations of AX; transforming into red-colored cyst cells, with increased longevity [13]. The unique ability of *Haematococcus* algae to accumulate high concentrations of natural AX is leveraged for industrial production.

### 1.2. Biological Activity of Astaxanthin

#### 1.2.1. Function of Astaxanthin in Lipid Bilayers: Antioxidant Activity and Impact on Physical Properties

**Figure 2:**
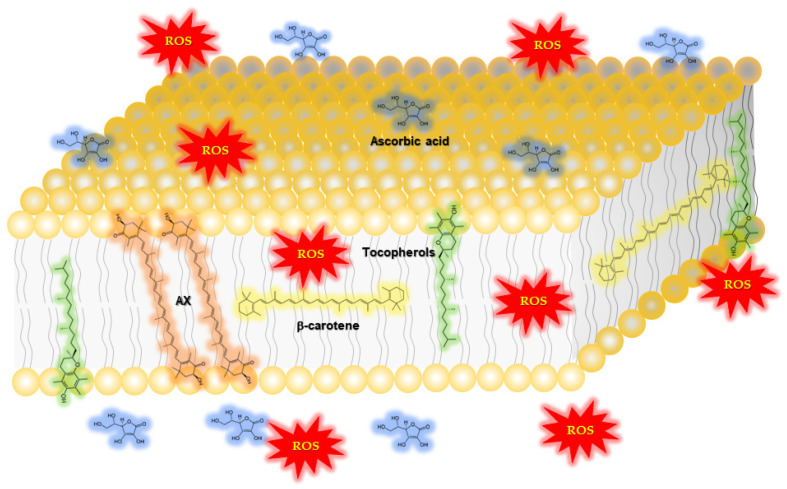
AX performs its antioxidant activity both inside and on the surface of the plasma membrane. Due to its strongly hydrophobic conjugated polyene structure and terminal polar groups, AX can exist both inside and on the surface of the phospholipid membrane. Therefore, AX is able to exert its effects against ROS both at the surface and inside of phospholipid membranes. On the other hand, β-carotene exerts its antioxidant activity only inside the phospholipid membrane. As for other antioxidants, ascorbic acid cannot exert its effect inside the phospholipid membrane, due to its high hydrophilicity, whereas tocopherols are relatively effective at the surface of the phospholipid membrane. This figure excludes the detailed structure of the cell membrane, including localization of different levels of lipids lipid rafts and proteins to avoid complications.

AX has antioxidant activity, a well-known characteristic of carotenoids. Aside from its ability to quench a number of reactive oxygen species (ROS) and reactive nitrogen species (RNS), and other free radicals, AX stands out among carotenoids due to its particularly strong singlet oxygen quenching activity [14,15,16]. AX is also well-known for strongly inhibiting the accumulation of lipid peroxides resulting from lipid peroxidation chain reactions [17,18]. In biological environments, AX has been detected in lipid droplets [19], cell membranes [20], or bound to proteins [18,21,22,23], due to its highly lipophilic properties. In addition, the structure of AX, like several other xanthophylls, it is thought to span across phospholipid bilayers that form biological membranes [24,25,26,27,28]. This is based, in part, on the observation that AX was able to quench or scavenge ROS, RNS and free radicals both in the interior and surface layers of lipid membranes (Figure 2).

The antioxidant activity of some carotenoids can shift to pro-oxidant activity depending on carotenoid concentrations, under conditions of high oxygen tension, or based on interactions with other compounds [29]. Therefore, carotenoids are categorized into three classes: (A) those without significant antioxidant properties; (B) those with good antioxidant, but also pro-oxidant properties; and (C) those with strong antioxidant and without any pro-oxidant properties. AX was categorized as class (C), whereas β-carotene and lycopene were identified as class (B) [29]. Therefore, AX is often described as a “pure antioxidant”. In fact, it has been demonstrated that AX, in contrast to β-carotene and lycopene, exhibited significant antioxidant activity and reduced lipid peroxidation in a liposomal model membrane [25]. When applied to biological membranes, AX may allow *Haematococcus* cyst cells to resist oxidative stress resulting from adverse environmental conditions [13,30]. AX may also exert a protective role in muscle cell membranes during the extreme physical exertion experienced by salmon, during migration from the sea to their spawning ground. Based on this scenario in salmon, AX has also been investigated as an intervention for oxidative muscle damage during and after endurance exercise [31]. Although it is still unclear whether the observed effects of AX are a result of its direct and/or indirect antioxidant activity, several clinical reports have shown that AX reduced oxidative stress markers in humans (Table 1).

Aside from the antioxidant effect of AX on membranes, AX and other carotenoids also changed the membrane dynamics of model membrane structures and microsomes [25,27]. The effect on membrane dynamics may be influenced by the properties of both (i) the carotenoid, and (ii) the membrane.

(i) With respect to the influence of carotenoid properties, it is known that xanthophylls increase the order of phospholipid membrane packing, and decrease alkyl-chain motion in the fluid phase. These effects are strongest for dipolar xanthophylls (i.e., AX), significantly weaker for monopolar xanthophylls (i.e., β-cryptoxanthin), and negligible for nonpolar carotenes (i.e., β-carotene) [51]. In addition to carotenoid polarity, the concentration of carotenoids in the membrane may also influence the dynamics.

(ii) Cell membranes are composed of a variety of lipids and many different proteins, whose distribution is not homogeneous. Therefore, although AX slightly increased membrane rigidity in microsomes, this effect may not be ubiquitous across all biological membranes. Membranes of different cell organelles have distinct lipid compositions, and characteristic regions within membranes may coalesce certain types of lipids to form defined regions called microdomains. Carotenoids may have characteristic distributions across different cellular organelles or membrane microdomains.

For example, membrane regions enriched in sphingolipids and cholesterol are called lipid rafts, which are defined as “*small (10–200 nm), heterogeneous, highly dynamic, sterol- and sphingolipid-enriched domains that compartmentalize cellular processes. Small rafts can sometimes be stabilized to form larger platforms through protein–protein and protein–lipid interactions*” [52]. Lipid rafts have increased membrane thickness as well as characteristic membrane dynamics, and they play very important roles in membrane protein signaling, and sorting through the secretory and endocytic pathways [52].

Generally, highly polar xanthophylls with hydroxyl groups are not predominant in lipid rafts; rather, they are enriched in the fluid-phase of phospholipid model membranes that are predominantly composed of unsaturated fatty acids. In contrast, low-polarity carotenes are localized in both types of membranes: the more ordered lipid rafts, and the more fluid membranes are rich in unsaturated fatty acids. Although the direct relationship between carotenoids and their distribution in membrane microdomains is still unclear, some carotenoids have inhibited the translocation of important membrane receptor proteins into lipid rafts (e.g., immunoreceptors) [53,54] or affected the function of lipid raft proteins via their antioxidant activity (e.g., rhodopsin) [51].

Cholesterol is another important modulator of membrane dynamics and function in lipid rafts and elsewhere. AX has been shown to interact with cholesterol by inhibiting the peroxidation of cholesterol to 7-keto-cholesterol better than other common carotenoids [55]. We also reported that after insulin administration, AX had an acute effect in a type of lipid raft called a caveolae, whereby AX modulated the association between an insulin receptor and its adaptor protein [56]. Although it is unclear whether this effect was due to AX’s antioxidant activity or other factors, AX acutely enhanced the insulin-dependent glucose uptake signaling via phosphatidylinositol 3-kinase (PI3K)/Protein Kinase B (Akt) activation. Simultaneously, when cytokines and free fatty acids were used to induce chronic ROS accumulation and insulin resistance in rat L6 myotubes in vitro, AX enhanced insulin sensitivity and PI3K/Akt activation by insulin [56]. Thus, AX has the potential to protect and to directly modulate important structures in biomembranes.

**Figure 3:**
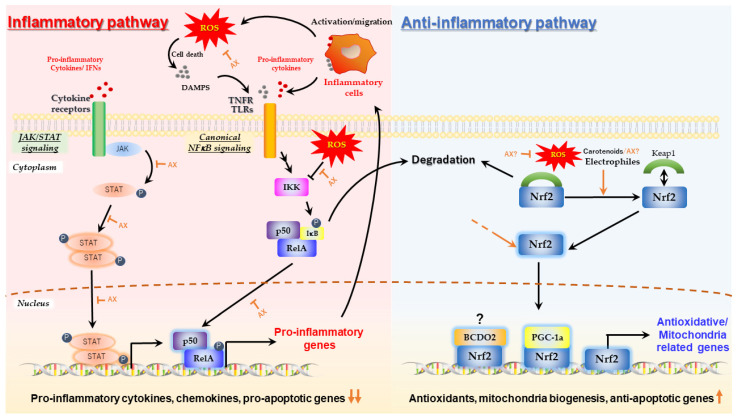
AX partially induces the antioxidant defense system while inhibiting the ROS-mediated inflammatory signaling pathway. AX inhibits ROS-mediated activation of canonical NFκB signaling and related signals such as JAK/STAT3. Consequently, the induction of pro-inflammatory cytokine gene expression is suppressed, resulting in attenuation of inflammatory signals. On the other hand, AX produces partial activation of Nrf2 via dissociation of Nrf2/Keap-1 by electrophiles, and/or other pathways. Consequently, antioxidant enzymes are induced and act in an anti-inflammatory function in vivo. Thus, AX suppresses the exacerbation cycle of chronic inflammation and shifts the cycle toward improvement. The regulation of these inflammation-related signaling pathways by AX involve a mixture of acute-phase responses to AX that result from ROS scavenging, modulation of phosphorylation and protein modifications related to the regulation of intracellular Redox balance, modulation of adaptor protein association with receptors, and the more chronic induction of gene expression mediated by these results. In this figure, lipid rafts and precise and detailed signal pathways are not shown to avoid complications. In particular, it has been reported that AX affects the points indicated by the orange arrows. This figure was adapted from the reference [70,71].

One of the most important physiological activities of AX, which is strongly associated with its antioxidant activity, is its anti-inflammatory activity in response to inflammation triggered by ROS-induced oxidative damage. Numerous studies have shown that AX inhibits canonical nuclear factor-kappa B (NFκB) signaling in response to oxidative stress via the inhibition of IKK oxidation, regardless of the source of ROS, cell types, or organ [31,57,58,59,60,61,62,63,64,65,66,67,68]. As a result, AX suppressed NFκB-mediated gene expression of pro-inflammatory cytokines such as IL-1β, IL-6, IL-8, iNOS or TNFα, thereby inhibiting the development of inflammation. AX is reported to inhibit the phosphorylation and nuclear translocation of STAT3 in the 7,12-dimethyl benz[a]anthracene (DMBA)-induced hamster buccal pouch (HBP) carcinogenesis model [69]. Therefore, it is likely that AX can act in an inhibitory manner on the JAK/STAT pathway, which is an inflammatory signaling pathway of cytokines such as IL-6, although there is little evidence that it works in the same way in all cells (Figure 3).

In conclusion, the antioxidant activity of AX exhibits potent antioxidant activity, and is able to inhibit ROS-induced damage, particularly in lipid membranes.

## 2. Mechanism by Which Astaxanthin Enhances Mitochondrial Energy Metabolism

### 2.1. Protective Effect of Astaxanthin on Mitochondria; Astaxanthin as a Mitochondrial Antioxidant

Many studies have observed a variety of cellular and molecular changes in response to AX treatment. Consequently, it can be difficult to determine which of these effects may be attributed to the direct mechanisms of action of AX, such as its direct antioxidant activity, or indirect downstream effects in response to chronic AX treatment. To address this, we focus below on the early changes resulting from acute exposure to AX.

The mitochondrion is an organelle that produces energy by electron transport chain (ETC)/oxidative phosphorylation, and oxygen is consumed in this process. Most of the oxygen molecules entering the ETC are reduced to water, but a significant amount escapes in the form of ROS byproducts [72]. AX can significantly inhibit the lipid peroxidation of biological membranes. It has also been reported that AX added to cultured cells was transported to the mitochondria [49]. Since most of the important components of the mitochondrial ETC are located within the inner membrane of mitochondria, AX is expected to protect mitochondrial membranes against oxidative damage caused by ROS. This is particularly relevant under conditions where ROS are overproduced, such as during conditions of metabolic stress caused by metabolic diseases and senescence [73,74,75]. For example, AX was reported to be nephroprotective in a mouse model of diabetes mellitus [76], and inhibit the generation of mitochondrial-derived ROS in human renal mesangial cells induced by hyperglycemic insults in vitro [68].

AX inhibited the damaging effects of mitochondrial overload, including resulting in reduced muscle damage in rodents after heavy exercise [31], as well as reduced oxidative modification of skeletal muscle proteins, and reduced inflammatory markers after treadmill exercise in mildly obese mice given a high-fat diet [77]. These results suggest that AX may protect mitochondria from oxidative damage caused by ROS production when mitochondria are overloaded under conditions of physiological stress.

To investigate the antioxidant effect of AX on mitochondria, Wolf et al., examined PC12 cells, which are highly responsive to oxidative stress. This report challenged PC12 cells with antimycin A (AnA), which inhibit Complex III triggering ROS overproduction, resulting in cytotoxicity. AX pre-treatment showed a time- and dose-dependent protective effect of AnA-treated PC12 cells, using sub-nanomolar amounts of AX [78]. This treatment did not cause cell death in HeLa or Jurkat cells, which have the ability to utilize the glycolytic pathway, bypassing the mitochondrial ETC. These results suggest that the addition of sub-nanomolar AX has a protective effect against oxidative damage caused by mitochondrial dysfunction in these cells. Interestingly, when organelle-localized redox-sensitive fluorescent proteins (roGFPs) were expressed in the cells, AX treatment did not change the level of cytoplasmic-reduced state under basal conditions or hydrogen peroxide (H_2_O_2_) treatment, but AX maintained a mitochondrial-reduced state under oxidative stress. In addition, when evaluated by the fluorescence of MitoSOX, a dihydroethidium (DHE)-derived mitochondrial-selective superoxide probe, there was no decrease in the production of mitochondrial-derived superoxide in the presence of AnA. The lack of evidence for the direct scavenging of AnA-mediated superoxide by AX in this in vitro experimental model may be due to superoxide being diffused into the aqueous space, while AX remains in the mitochondrial inner membrane. Despite not being able to observe the direct antioxidant activity of AX in this model, AX has exhibited physiological antioxidant activity or other physiological activities in a number of other studies, as will be discussed in later sections. In relation to that consideration, although the addition of AX did not increase the membrane potential of basal cells, it was useful in maintaining the membrane potential, which gradually decreased with incubation. Taken together, these results suggest that although AX does not inhibit ROS formation, it could be effective in improving mitochondrial function by neutralizing ROS to curtail the downstream effect on mitochondrial membranes.

In a recent report from another group, skeletal muscle cells (Sol8 myotubes) derived from mouse soleus muscle were challenged [79] by the addition of succinate, a substrate of Complex II and AnA that triggers the accumulation of ROS. ROS generated in the cells were observed using a fluorescent whole-cell superoxide probe (DHE), following the addition of AnA. Ax decreased the ROS-induced fluorescence in a concentration-dependent manner. Mitochondrial membrane potential was evaluated using JC-1 dye, which accumulates in mitochondria in the presence of mitochondrial membrane potential. Using JC-1 as an indicator of mitochondrial health and membrane integrity showed that the addition of AX alone did not change the basal mitochondrial membrane potential, but did inhibit the decrease in membrane potential resulting from AnA-induced ROS accumulation. Additional studies examined the ability of AX to protect mitochondrial membranes under various conditions triggering oxidative stress. Another study reported that AX helped protect mitochondrial respiratory chain activity against Fe^2+^-induced lipid peroxidation in mitochondria that were isolated from vitamin E-deficient rats [80]. AX also had a protective effect against ROS-mediated angiotensin II (Ang II)-induced mitochondrial dysfunction in vascular smooth muscle cells (VSMCs), and normalized mitochondrial respiratory parameters in the presence of ROS [80].

In response to oxidative stress, mitochondria can initiate programmed cell death, also known as apoptosis. Oxidative stress disturbs intracellular Ca^2+^ homeostasis, resulting in excessive Ca^2+^ efflux from the endoplasmic reticulum and an influx into mitochondria, which subsequently triggers mitochondrial membrane permeabilization, loss of mitochondrial membrane potential, and the release of mitochondrial pro-apoptotic factors [81]. It has been widely reported that AX prevents the ROS-induced Ca^2+^ influx into mitochondria, protects against mitochondrial dysfunction, and inhibits apoptosis [82,83,84,85,86,87,88]. The role of AX in modulating mitochondrial-mediated activation of apoptosis is beyond the scope of this review. However, the authors acknowledge that there has been extensive research on this subject, which merits its own dedicated literature review.

Although the effects of AX differ slightly depending on the cell type, detection system, and mitochondrial substrate and condition, all reports have indicated that AX has a protective effect on mitochondria, especially on membrane components. Thus, the antioxidant effects of AX on membranes are not isolated to a single cell strain.

Summarizing these reports, it was suggested that AX could somehow act to maintain and protect the integrity of the mitochondrial ETC and oxidative phosphorylation against oxidative stress. However, the cells used in these studies underwent relatively long-term AX treatments, possibly to overcome the slow intracellular uptake of AX. Thus, it is unclear whether the observed mitochondrial protective effects were due to the direct antioxidant action of AX, induction of antioxidant enzymes via the Nrf2-Keap1 pathway, or remodeling of mitochondria-related genes. Therefore, the presence of AX-mediated regulation of mitochondria-related gene expression and its putative mechanisms are presented in the following sections.

### 2.2. Aggressive Enhancement of Mitochondrial Activity and Metabolism via Gene Expression by Astaxanthin

We, among others, have shown that AX improves glucose and lipid metabolism and muscle strength [77,84,89,90,91,92], mainly by correcting abnormal gene expression or protein modification in the mitochondria, which is altered during oxidative injury [77,93]. These effects are mainly attributed to the antioxidant effects of AX.

In fact, ROS production due to decreased activity of the mitochondrial ETC is thought to be involved in energy overload and metabolic disturbances [73,94]. Paradoxically, it is widely recognized that at physiological levels, ROS generated from mitochondria are also beneficial in improving metabolism in response to exercise [95,96,97]. Unfortunately, it is practically difficult to distinguish between the physiological levels of ROS and levels resulting in oxidative stress. Furthermore, the pharmacological effects of AX were considered too complicated to be explained by only its antioxidant effects as a single compound. Thus, the authors considered other mechanisms of action of AX outside of its antioxidant action [92].

#### 2.2.1. Nrf2 Pathway

Nuclear factor erythroid 2-related factor 2 (Nrf2), is a transcription factor that plays an important role in maintaining redox status and in modulating inflammation [70], as well as in mitochondrial biogenesis and function [98]. Nrf2 interacts with target genes at DNA binding sites called antioxidant response elements (AREs). Nrf2 activity is modulated by the Kelch-like ECH-associated protein 1 (Keap1)/Nrf2, epigenetic DNA elements, PI3K/Akt pathway, and other transcription factors. Nrf2 dissociates from Keap-1 and is translocated from the cytoskeleton in the cytosol into the nucleus, where it can induce gene expression in response to ROS. Dissociation of Nrf-2 from Keap-1 is facilitated by ROS and strong electrophilic compounds, like polyphenols and isothiocyanates [70].

Early studies of carotenoids showed that lycopene significantly activated Nrf2 via Nrf2/Keap1 dissociation [99], and later it was shown that the degradation products of lycopene were the main active forms [100]. Lycopene metabolite is indeed a strong electrophilic compound, and could be considered an inducer of Nrf2. The impact of AX on the Nrf2 pathway for various cell types and disease models has been described in other good review papers [71]. It should be noted, however, that it is unclear whether this is a canonical pathway via dissociation of Keap1 or the result of some indirect non-canonical activation pathway. Indeed, AX increases the expression of Nrf2 in certain pathological models and in certain tissues [92,101,102]. Unfortunately, most studies investigating the effect of AX on Nrf2 activation did not examine downstream gene expression, including the targets of Nrf2, such as the glutamate-cysteine ligase catalytic subunit gene (*Gclc* in rodents, *GCLC* in human) and the NAD(P)H:quinone oxidoreductase-1 gene (*Nqo1* in rodents, *NQO1* in human). Only heme oxygenase-1 gene (*Hmox1* in rodents, *HMOX1* in human) was used as a reporter gene, and was not confirmed by loss-of-function studies to determine whether Nrf2 was truly involved in its AX-induced activation.

To address the question of the Nrf2-mediated activation of antioxidant enzymes in response to AX, we used obese mice to evaluate the expression of antioxidant enzymes downstream of Nrf2 and other genes in various tissues, and found that even in epididymal adipose tissue, which was most affected by oxidative stress, gene expression of several Nrf2 targets was altered, but there was no significant change in the gene expression status of *Gclc* or *Nqo1* ([92] and unpublished data). An important finding was that, when bone marrow-derived macrophages (BMDMs) isolated from wild-type and Nrf2-knockout mice were stimulated with lipopolysaccharide (LPS), AX reduced the accumulation of intracellular ROS, regardless of genotype. Thus, Nrf2 is unlikely to be involved in the reduction of intracellular ROS by AX [44].

Therefore, these results were confounding effects of other transcription factors, such as the peroxisome proliferator-activated receptor γ coactivator-1 (PGC-1α) [98], and it is doubtful whether this was mediated by the Nrf2/Keap1 pathway. In other words, we cannot deny the possibility that this is the result of enhancements in gene expression due to activation of the PGC-1α/Sirtuins pathway by AX (as described in the next Section 2.2.2.) and that Nrf2 is transferred to the nucleus as a result of oxidative stress rather than by the action of AX by the canonical Nrf2/Keap1 pathway.

In addition, very recently, it was also reported that mouse carotene-9′,10′-oxygenase (BCDO2 also called BCO2, details of the function of this carotenoid-substrate enzyme are described in Section 2.2.3 and Section 2.2.6) is a functionally palmitoylated enzyme that, upon binding to xanthophylls in the mitochondria, can be translocated into the nucleus via depalmitoylation. Once in the nucleus, it may bind to AREs, possibly in association with other transcription factors such as Nrf2, and then regulate downstream gene expression [103]. It has been reported that mice with whole-body knockout of BCDO2 function developed metabolic dysfunction derived from the peripheral and hypothalamus, even when fed a diet thought to be free of carotenoids. Importantly, failure of gene expression related to the antioxidant response, such as Nrf2, was observed frequently in the knockout mice used in these studies [104,105,106]. In conclusion, although the level of influence of AX on this pathway is not known, it is suggested that carotenoids may activate Nrf-2 in a different way to the commonly known Nrf2/Keap1 pathway (Figure 3.).

#### 2.2.2. Nuclear Receptors 

In rodents and primates, including humans, obesity caused by a high-fat diet is believed to induce insulin resistance, deteriorate glucose and lipid metabolism, and induce metabolic syndrome and type 2 diabetes (T2DM). In contrast, it has also been reported that, in a high-fat diet, skeletal muscle mitochondria and their component proteins are increased, likely as a compensatory mechanism, causing mitochondrial dysfunction [94]. It is strongly suggested that oxidative stress due to mitochondrial dysfunction is also involved in insulin resistance in adipose tissue and liver [107]. It has been reported that insulin resistance could be improved by AX [37,90,108]. Although most anti-diabetic drugs target the liver or adipose tissue for their pharmacological action, research has shown in a hyperinsulinemic-euglycemic clamp study in obese mice that AX exerts its function not in the liver, but in skeletal muscle and adipose tissue [92]. The skeletal muscle is the largest glucose metabolizing organ in the whole body, and has plasticity, responding to both exercise quality and quantity [109]. When we looked at the gastrocnemius muscle in AX-administrated mice, we found that gene expression was strongly altered in favor of glucose and lipid metabolism with or without obesity. This resulted in remodeling muscles to increase slow twitch fibers containing more mitochondria and blood vessels. This change in the quality of the skeletal muscle improved the endurance of the mice, which was consistent with other reports [77,91]. Possibly, these changes may indicate that the reported effects of AX on capillary regression in immobilized muscle atrophy may be due, in part, to effects other than the antioxidant activity of AX [110]. Furthermore, the expression of mitochondria-related transcription factors was altered in this skeletal muscle. These effects were mainly found in the gastrocnemius muscle, with smaller changes in other skeletal muscles (unpublished data). Of particular interest was the upregulation of gene expressions of a series of members of the peroxisome proliferator-activated receptor family and estrogen receptor-related family of genes such as *Ppargc1a, Ppargcb1*, *Perm1*, *Ppara*, *Esrra* and *Esrrg*. Interestingly, the gene expression of mitochondria-associated Sirtuins was also significantly increased. It has been reported that changes in the muscle expression of these genes can lead to enhanced lipid utilization, vascularization and improved insulin resistance in obesity [111,112,113,114,115] (see review [116]). In addition, 2-thiobarbituric acid reactive substances (TBARS), a marker of oxidative stress, were unchanged from the control mice, and there were no systematic changes in the expression of inflammatory cytokine genes, suggesting that they probably did not depend on antioxidant activity.

Considering this, it is possible that AX and its derivatives directly regulate nuclear transcription factors as ligands. For example, AX is known to regulate the gene expression of peroxisome proliferator-activated receptor (PPAR) family members, and is often recognized as a ligand [117]. Actually, it was revealed that AX bound to PPARγ by CoA-BAP assays in a dose-dependent manner, acting as partial inhibitors to regulate parts of the genes of PPARγ targets in in vitro studies, using PPARγ reporter assays in adipocytes and macrophages [118]. It has been reported that AX regulates the gene expression of ATP-binding cassette transporters (ABC) A1 and G1, which are key molecules in cholesterol efflux from macrophages, the first step in reverse cholesterol transport, a major anti-atherosclerotic property of high-density lipoprotein (HDL). This effect is mainly due to activation of the liver X receptor (LXR) complexes with PPARγ or other nuclear receptors, such as all-trans retinoic acid receptors (RARs) and retinoid X receptors (RXRs), then transcriptional regulation by binding to LXR-responsive elements. Intriguingly, when a human ABCA1/G1 promoter–reporter assay was performed, AX activated both promoters with or without LXR-responsive elements, indicating LXR-independence in these activations [119]. This raises the possibility that AX, or its metabolites, partially bind to nuclear receptors such as RARs, RXRs, and PPARs, but not their full activation (such as full-agonist/antagonist), thus partially regulating their activity (such as partial agonist/antagonist). Unfortunately, there is currently no clear evidence for binding to nuclear receptors.

Apocarotenoids, the major metabolites of carotenoids by BCDO2 and oxidation, have also been shown to have effects on these nuclear receptors [120]. There are a few pieces of information available to shed some light on this putative pathway. Apo-canthaxanthinoic acids are metabolites of canthaxanthin that possess an AX-like 4-keto group. One canthaxanthin metabolite, 4-oxoretinoic acid, significantly enhances connexin 43 mRNA stability by binding to its 3′-UTR, which upregulates the expression of this component of gap junctions that mediates intercellular communication. Moreover, 4-oxoretinoic acid also activates retinoic acid-beta2 receptor (RXRβ2) to stimulate gap junction communication [121]. Regarding AX, it is known that derivatives of AX regulate the expression of connexin 43, and that AX itself enhances the expression of connexin 43 [122,123], although connexin 43 activity is affected by phosphorylation-mediated modifications by AX dose, dependently [124]. Since 3-hydroxy-4-oxo-β-ionone and its reduced form 3-hydroxy-4-oxo-7,8 dihydro-β-ionone were found in human plasma as metabolites of AX, they may be responsible for mediating this activity [125]. These results suggest that AX may also be a partial agonist of RARs and RXRs, although it is much weaker than all-*trans* retinoic acid.

Interestingly, we have also shown an effect of carotenoids, including AX, on retinoic acid-related orphan receptor gamma *t* (RORγ*t*) as a receptor mediating CD4^+^ T cell differentiation into Th17 cells. In summary, when naïve mouse T cells were treated with IL-1β, IL-6, IL-23, and anti-IFN-γ antibodies to induce pathogenic Th17, AX suppressed pathological Th17 maturation, and reduced the gene expression of IL-17A, which plays an important role in the development of pathogenicity. However, it does not affect the expression of IL-17F, which is involved in intestinal biological defense (unpublished data, patent publication No. JP2020117465A). In other reports of Th17 induction by addition of TGF-β and IL-6, including non-pathogenic Th17, only fucoxanthin among various carotenoids exhibited significant inhibition of secretion of IL-17, which may be found both as IL-17A and IL-17F [126]. Focusing on the differences between the two studies, our study was more affected by the RORγ*t* induction of Th17 cells, suggesting that perhaps carotenoids or their derivatives, including AX, can function as antagonists of RORγ*t*. The activity itself is probably weak, but it may have some impact on chronic inflammation and immunity in tissues with high exposure, such as in the intestine.

In mice, AX significantly accumulated in adipose tissue and liver, indicating that the activities shown above probably contribute to the pharmacological effects of AX on nuclear receptors [108,127]. However, it is necessary to consider species differences in the effects on nuclear receptors, especially the PPAR family. For example, it is known that AX and its metabolites induce cytochrome P450 (CYPs), such as CYP1A1, CYP1A2, CYP3A4 and CYP2B6 in rodent hepatocytes, probably via PPARα activation by AX. However, this effect requires several tens fold higher concentration in human hepatocytes, compared with that in rats [125]. Furthermore, since the beneficial effects of AX on metabolisms and skeletal muscle function have been shown in human clinical trials (Table 1), the actual contribution of PPARs might be minor.

It is suggested that there may be mechanisms of action that are less sensitive to species differences, such as specific antioxidant activities and other mechanisms. Based on this idea, we investigated the mechanism of action; as one of targets of AX we have identified “***AMP-activated protein kinase***” (AMPK) [92].

#### 2.2.3. AMPK/Sirtuins/PGC-1α Pathway

**Figure 4:**
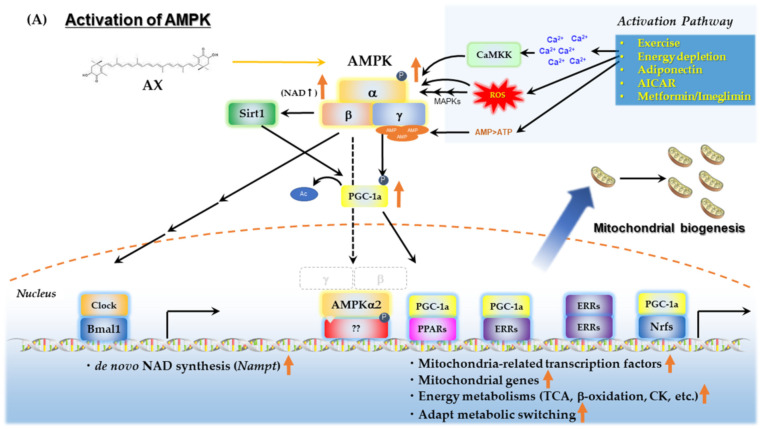

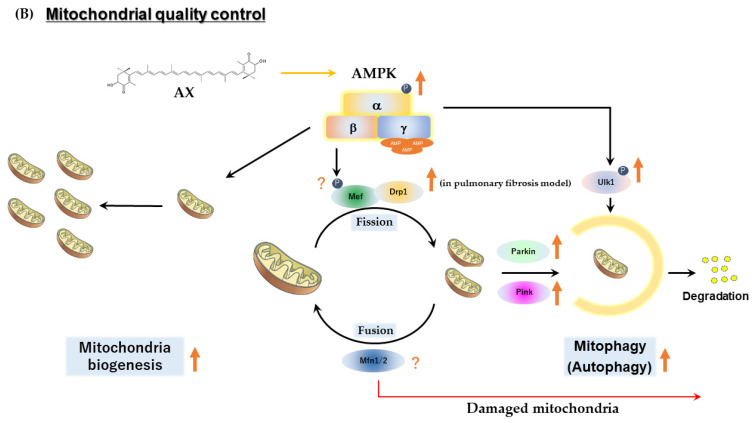
AX regulates various mitochondria-associated metabolic pathways, mitochondrial biogenesis and its quality control via AMPK activation. (**A**) AMPK is activated by exercise, energy depletion, or certain active drugs (e.g., AICAR, adiponectin, metformin and imeglimin) by (1) increased Ca2+ influx; (2) direct modification by ROS and activation of MAPKs; and (3) increased AMP/ATP ratio. Activated AMPK induces activation of PGC-1α and related gene expression, leading to enhanced energy metabolisms, adapted metabolic switching, and increased mitochondria biogenesis. Furthermore, AMPK regulates gene expression of Nampt and promotes de novo synthesis of NAD+ in the cell. As a result, it enhances the activity of Sirtuins and further enhances the activity of PGC-1α. Thus, AMPK/Sirtuins/PGC-1α forms a positive feedback loop in their actions. (**B**) AMPK contributes to mitochondrial quality control; AMPK not only enhances mitochondrial biogenesis, but also regulates mitochondrial fission and fusion via phosphorylation of Mef, and induces mitophagy in autophagosomes via the phosphorylation of Ulk-1 for the impaired mitochondria. AX activates AMPK. In particular, it has been reported that AX affects the points indicated by the orange arrows. In this figure, precise and detailed signal pathways are not shown, to avoid complications. This figure was adapted from the reference [116,133,134].

AMPK is a key sensor of cellular energy status present in essentially all eukaryotes. It is a heterotrimer comprising a catalytic α subunit and regulatory β and γ subunits [128]. AMPK plays a crucial role in energy metabolism, including lipid, glucose and protein metabolism, and is also important for mitochondrial biogenesis and quality control. In recent years, AMPK has received much attention for its important role as a target of metformin, thiazolidinediones, and exercise therapy for the treatment of T2DM and related metabolic diseases [129]. In skeletal muscle, AMPK and SIRT1/PGC-1α work together to mediate metabolic adaptation during fasting and exercise [130]. These reciprocal enhancements of activity result from the direct induction of *Pgc1a* gene expression by AMPK, the enhancement of activity via deacetylation of PGC-1α by SIRT1, and the increase in intracellular amounts of nicotinamide adenine dinucleotide (NAD^+^) by the induction of *Nampt* gene expression by AMPK. These unique interactions are discussed in detail in another good review [116]. Previous studies have shown that AX increases the levels of PGC-1α in skeletal muscle [89,131]. To determine if upregulation of PGC-1α in response to AX was mediated by AMPK, we examined PGC-1α expression using a mouse skeletal muscle cell line (C2C12 cells), following the knockdown of AMPKα1/2 expression. We observed that AMPKα1/2 knockdown abolished the increased expression of PGC-1α in response to AX, indicating that AX directly stimulates AMPK [92]. This suggests that the effect of AX in upregulating PGC-1α levels in skeletal muscle occurs via an AMPK-dependent pathway (Figure 4A) [92].

#### 2.2.4. AX Contributes to Mitochondrial Quality Control

AX also probably has a beneficial effect on mitochondrial quality control, mainly through AMPK activation. It has been reported that AX can prevent pulmonary fibrosis by promoting myofibroblast apoptosis through dynamin-1 like protein (Drp1)-mediated mitochondrial fission [132]. In this report, AX enhanced the expression of Drp1. Furthermore, AMPK phosphorylates and activates mitochondrial fission factor (MFF), which associates with Drp1, leading to mitochondrial fission [133]. These reports use experimental models with mitochondrial dysfunction, such as cancer cells, which describe a beneficial aspect of AX mitochondrial quality control. In skeletal muscle, Drp1 is upregulated during acute phase exercise where mitochondrial fission is induced. In addition, Drp1 may play an important role in the processing of exercise-impaired mitochondria, since Drp1 deficiency reduced muscle endurance and running performance, and altered muscle adaptations in response to exercise training [134]. On the other hand, AX has a protective effect on mitochondria against heat stress and Ang II-induced mitochondrial dysfunction, at which time it normalizes the upregulation of Drp1 gene expression caused by the damage [83,135]. It has also been reported that AX activates autophagy and inhibits apoptosis in *Helicobacter pylori*-infected gastric epithelial cell line AGS via AMPK-mediated phosphorylation of Unc-51-like autophagy-activating kinase 1 (Ulk1) [136]. In addition, during AngII-induced mitochondrial damage to VSMCs, AX treatment resulted in the mitophagy-mediated induction of Parkin, PTEN-induced kinase 1 (Pink1) and the activation of autophagosomes [83]. In addition, as will be explained in Section 2.2.5, AX induces the gene expression of *sirt-3*, probably via ERRα or ERRγ and PGC-1α. Sirt-3 also plays a crucial role in mitochondrial dynamics and contributes to mitochondrial quality control [137]. Collectively, the quality control for dysfunctional mitochondria by AX seems to be achieved by AMPK and related signaling pathways (Figure 4B).

#### 2.2.5. Is the AMPK-Activating Effect of AX Independent of Its Antioxidant Effect?

It is well known from large-scale epidemiological studies that moderate exercise increases energy expenditure and improves obesity, thereby preventing and improving T2DM [138,139,140,141,142]. Interestingly, as an epidemiological intervention study, the actual incidence of T2DM was followed up for about three years in thousands of subjects diagnosed with high risk of developing T2DM, and it was found that the incidence of T2DM was significantly reduced when metformin, an oral biguanide-derived antidiabetic and AMPK activator, was taken before the onset of the disease (from *Diabetes Prevention Program* (DPP) [143]). Collectively, moderate exercise-dependent or -independent activation of AMPK is a very useful strategy for preventing the incidence of T2DM and improving energy metabolism.

The beneficial effects of moderate exercise on the metabolism are partially mediated by ROS [95,96,97], which involves the activation of AMPK by a physiological level of mitochondrial ROS [144,145,146,147]. Exercise-induced activation of AMPK depends on the physiological mechanisms of muscle contraction, *e.g.,* energy depletion, increasing influx of Ca^2+^, activation of MAPK pathways (e.g., p38) by mitochondrial ROS, and direct oxidative modification of AMPK (Figure 4A). Therefore, administration of antioxidants may have an adverse effect on exercise therapy for glucose or lipid intolerance in T2DM and metabolic syndrome. In fact, the chronic administration of certain antioxidants counteracts the glucose tolerance that is improved by exercise therapy [148] and training-induced adaptations in endurance performance [149]. Thus, it is still controversial whether many other antioxidants including AX can be useful in improving exercise performance [150]. AX may be beneficial for skeletal muscle damage after high-intensity endurance exercise, as shown by Aoi et al. [31]. However, the influence of AX on mild to moderate intensity aerobic exercise, used to address ROS levels in T2DM and obesity, is less clear.

Recently, it has been reported that the antioxidative hepatokine, selenoprotein P (SeP) caused a physiological effect in skeletal muscle called “exercise resistance”. Exercise resistance decreases the therapeutic effects of exercise as an intervention for glucose intolerance by inhibiting the ROS-mediated activation of AMPK [151]. Exercise resistance has also correlated with plasma concentration of SeP in humans. Therefore, it is necessary to carefully judge whether the chronic administration of AX will cause exercise resistance. Aoi et al. have shown, in part, that AX further enhanced lipid utilization in obese mice with daily exercise training [77]. We also evaluated glucose tolerance in obese mice that received daily exercise, and found that AX, together with exercise, improved glucose tolerance in an additive manner [92]. In further in vitro cell studies using C2C12 myotube, we evaluated the phosphorylation of Thr^172^ in AMPKα by the addition of H_2_O_2_, an ROS that activates AMPK. When comparing the effect of antioxidants on ROS-mediated AMPK phosphorylation, the H_2_O_2_ scavenger, *N*-acetylcysteine (NAC), inhibited AMPK phosphorylation, whereas AX did not [92]. Therefore, it was concluded that AX does not interfere with the beneficial effects of exercise therapy. This result may reflect that AX is less effective in neutralizing H_2_O_2_, compared with other kinds of ROS. Another explanation may be that AX and AMPK may be localized at different sites in the cell.

It has been reported that the phosphorylation of Thr^172^ residue of AMPKα is dephosphorylated by protein phosphatase 2A (PP2A), a serine-threonine protein phosphatase, resulting in the inhibition of AMPK activity [152]. We also observed that AX increases PP2A phosphorylation and decreases its activity at the basal state, which is concomitant with enhancement of insulin-induced Akt phosphorylation in L6 cells [37,153]. As PP2A itself is of low sensitivity to the ROS, it appears that the ROS-related activation of PP2A requires a cofactor such as the Src kinase family, which is also known to be resident at lipid raft, and activated under the change in redox balance. AX may activate this kinase through modification of the redox balance at the membrane. Therefore, the antioxidant activity of AX may also have an effect on the activation of AMPK by ROS, although this is complicated by the localization and type of ROS.

There are also two types of AMPKα subunit: AMPKα1, which is localized in the cytosol; and AMPKα2, which is localized in the mitochondria or nucleus, and it is still unknown which one can be activated by AX. The functions of these two proteins are distinct. It has recently been shown that AMPKα2, but not AMPKα1, activates the transcription of the PGC-1α gene by translocating into the nucleus when it is activated [154]. The activation of AMPKα2 is required to maintain skeletal muscle integrity [155]. AMPKα1 contributes to the middle stage of myogenesis, and rather regulates the ACC-mediated mitochondrial incorporation of fatty acids and autophagy [154]. Furthermore, the activation of AMPKα2 contributes to inducing the gene expression of ERRα and ERRγ, although the response varies by tissue [156]. ERRα and ERRγ cooperate with PGC-1α to regulate the expression of a group of genes involved in mitochondria-related genes, muscle fiber, angiogenesis, hypoxic response and myogenesis, which is beneficial for the energy metabolism in mitochondria [157,158,159]. Moreover, in the skeletal muscles of AX-treated obese and lean mice, the gene expression levels of Sirtuins such as *Sirt1* and *Sirt3* were upregulated, and *Nampt* was also upregulated, which is closely related to AMPK activation [92,160]. Furthermore, intracellular NAD^+^ levels were significantly elevated when C2C12 myoblast cells were treated with AX, compared with the solvent vehicle group. Therefore, it is likely that AX activates Sirtuins via activating de novo synthesis of NAD^+^ (unpublished data, see Supplemental Material and Supplementary Figure S1 for details). It is reasonable to conclude that this is the result of the activation of both AMPKα1 and AMPKα2, because the activation of AMPKα1 by AX enhances the phosphorylation of ACC, resulting in increased lipid incorporation, and the activation of AMPKα2 also causes changes in gene expression in skeletal muscle. However, the intensity of activation was significant only in skeletal muscles with high plasticity or with mixed muscle fibers, such as the gastrocnemius. Meanwhile, skeletal muscle mass was not significantly increased (unpublished data), suggesting that it is reasonable to position AX as a partial exercise mimetic [92]. Collectively, we believe that AX administration in skeletal muscle forms a positive feedback loop via AMPK activation on mitochondrial biogenesis and energy metabolism by: (1) activation of Sirtuins by regulating NAD+ levels; (2) activation of PGC-1α by Sirtuins; (3) induction of gene expression of Sirtuins by increasing ERRs expression; and (4) their concerted action (Figure 4A).

In addition to the above, there is another important mechanism of AX mitochondrial activation, including AMPK activation, which is believed to be through the action of adiponectin and its receptors. It has been reported that the administration of AX significantly increases the amount of adiponectin in the blood, and its gene expression in adipose tissue, in obese animals [90,92] and humans [161,162,163]. Adiponectin is an adipokine with beneficial aspects secreted by smaller adipocytes, and its gene expression is regulated by PPARγ, which plays a role in the beneficial effects of its agonist, antidiabetic drug thiazolidinediones [164,165]. Although AX is a partial modulator of PPARγ [118], it does not seem to have any inhibitory effects on adiponectin gene expression. The receptors of adiponectin are AdipoR1 and AdipoR2, which are expressed at different levels in different tissues, and are involved in the regulation of glucose and fatty acid metabolism, mainly through the activation of Ca^2+^ signaling, AMPK/SIRT1, and PPARα signaling pathways [165]. It has been reported that AX has an inhibitory effect on Ca^2+^ signaling, which is mainly involved in ROS [83,85,86,87,88], but its effect on Ca^2+^/calmodulin-dependent protein kinase β (CaMKKβ), which is involved in the activation of AMPK [166] is still unknown. Thus, to summarize what is currently known, oxidative stress decreases the amount of adiponectin and its receptors. In contrast, AX prevents or increases the amount of adiponectin and its receptors, possibly leading to the activation of AMPK.

In recent years, mitochondria have been implicated in a diverse number of processes related to aging, including senescence, inflammation, and further age-related functional impairment of tissues and organs [167,168]. In skeletal muscle, the relationship between mitochondrial dysfunction and insulin resistance during aging is confounded by many factors, suggesting some association, although this is complex. For example, age-related mitochondrial dysfunction raises the level of ROS release from mitochondria, which induces phosphorylation of serine in IRS proteins and disturbs insulin signaling, resulting in insulin resistance [169,170,171]. As shown in Section 1.2.1, AX regulates insulin signaling. We have shown that AX inhibits the serine phosphorylation of the IRS-1 protein by ROS. However, we cannot eliminate the possibility that this effect is acute and unrelated to the mitochondria-mediated response [56]. Additionally, as shown many times, we have reported that AX potentially improves mitochondria function via AMPK/Sirtuins/PGC-1α pathway [92]. Furthermore, AX potentially elevates the level of NAD^+^ in cells (supplementary Figure S1). In recent years, importantly, it has been revealed that increasing the intracellular NAD^+^ concentration may improve age-related mitochondrial dysfunction and insulin resistance, which has attracted researcher’s attention [172,173,174].

Attempts to increase NAD^+^ levels in vivo, such as supplementation with nicotinamide mononucleotide (NMN), an intermediate in the biosynthesis of NAD^+^, have potentially ameliorated age-related insulin resistance and improved mitochondrial function (see review [173]). Regarding the beneficial effects of AX on age-related metabolic changes, the authors preliminarily evaluated the effects of AX on insulin resistance and glucose intolerance with aging using male C57BL/6J mice (unpublished data, see Supplemental Material and Supplementary Figure S2 for details). In mice, glucose intolerance and insulin resistance occur with age, and later, compensatory islet expansion and hyperinsulinemia, although this is still controversial depending on feeding conditions, genetic background, and sex [175,176,177,178,179,180]. AX was administered for about one year, which corresponds to middle age when metabolic abnormalities would occur in humans [181]. Continuous administration of AX to mice on a normal chow diet significantly improved age-related insulin resistance and glucose intolerance.

It is also well known that prediabetes and Alzheimer’s disease both increase in prevalence with age. Recently, a direct connection was revealed between peripheral hyperinsulinemia, as found in prediabetes, and age-related neurodegeneration and cognitive decline, such as Alzheimer’s disease [182]. The role of AX in cognitive function is beyond the scope of this review. However, the authors acknowledge that there has been extensive research on this subject, which merits its own dedicated literature review. In human clinical trials and animal models, AX has been shown to be beneficial in cognitive decline associated with aging [183,184,185,186,187,188]. Although most of the discussion of the mechanism of actions is based on the antioxidant effects of AX, the authors would like to suggest that this should be expanded to include mechanisms such as described in this section.

Based on these findings, as a strategy for preventing age-related diseases and extending lifespan, in addition to supplementation with NAD+ intermediates such as NMN, AX may also have beneficial effects on age-related diseases. Even in clinical trials, AX has been shown to be beneficial in extending walking distance and increasing muscle strength in the elderly [189,190,191]. (Details are given in Table 2). Therefore, AX is also an attractive potential candidate for anti-aging. The potential of AX for life extension is also shown in Section 2.2.6.

To summarize this section, AX treatment has been seen to: (i) significantly ameliorate insulin resistance and glucose intolerance through AMPK activation; (ii) stimulate mitochondrial biogenesis in the muscle; (iii) enhance exercise tolerance and exercise-induced fatty acid metabolism. This mechanism of action is similar to that of imeglimin, which has just been launched in Japan for an anti-diabetic drug, although the target molecules are probably different [192].

#### 2.2.6. Direct Effects of Astaxanthin on Mitochondria: Actions beyond Its Antioxidant Activity

It is still unclear how AX directly affects mitochondria, other than through its antioxidant activity, including its redox-mediated PP2A inhibition of AMPK activation. However, looking at the pharmacological efficacy of AX shown in above section, it is very difficult to explain its physiological activities by only referencing AX’s antioxidant activity. It is possible that AX may physically affect mitochondrial membrane dynamics due to its rigid and long conjugated double bonds, and its terminal polar groups. AX may affect the function of mitochondrial membranes and membrane proteins (like the ETC) by a mechanism similar to that described for insulin signaling, whereby AX modulated the association of adaptor proteins with insulin receptors in the plasma membrane (as shown in Section 1.2.1). To support the notion that AX modulates mitochondrial membrane proteins, we can refer to a few reports on the effects of AX on the mitochondrial respiratory chain proteins.

Wolf et al., evaluated the activity of the mitochondrial respiratory chain in HeLa cells and found that the addition of AX increased oxygen consumption in the basal condition, but did not observe any significant changes in the maximal oxygen consumption in the presence of the mitochondrial uncoupler, oligomycin [78]. Therefore, the ratio of baseline to uncoupled oxygen consumption was significantly higher in the presence of AX. However, this effect may also be mediated by changes in gene expression in response to AX administration to the cells.

One report took a different approach to the study of mitochondrial protection, using rat heart mitochondria. They found that AX inhibited the opening of mitochondrial permeability transition pores (mPTPs), which are a large conductance channels formed after the overload of Ca^2+^, or in response to oxidative stress. AX treatment in this model also slowed down the mitochondrial swelling associated with the opening of mPTPs [82,215].

When glutamate and malate were used as substrates in isolated rat heart mitochondria and AX was added at a relatively high concentration of 5 μM, decreased State 4 respiration with increased ADP-dependent State3 respiration conjugated with ATP synthesis was observed. In fact, the respiratory control index (RCI), which indicates the effectiveness of the mitochondria in promoting oxidative phosphorylation and coupling between oxygen consumption and ATP production, also increased with AX treatment [215].

We also conducted a study using mouse liver mitochondria to evaluate the direct impact of AX on mitochondria (unpublished data, see Supplemental Material and Supplementary Figure S3 for details). In this study, when 5 μM of AX was added to isolated mitochondria from liver tissue of male C57BL/6J mice, the oxygen consumption of States 4 (and also States 2) was significantly increased by addition of AX when succinate (which enters the respiratory chain at Complex II) was used as a substrate. However, there was no change in oxygen consumption when glutamate and malate were used as substrates (enter the respiratory chain at Complex I), while the RCI decreased. This effect of AX was completely abrogated by the addition of rotenone, an inhibitor of Complex I, suggesting that AX acts downstream of Complex I. There was no effect of AX on State 5 respiration with the addition of FCCP, a strong ETC uncoupler. The results from the mouse liver tissue suggest that AX may directly regulate the flow of electrons in the pathway between Complex II and Complex III via the quinone pool, which is consistent with findings referred to as the reverse electron transfer (RET) [72]. Thus, AX itself may be a mild uncoupler, perhaps via RET, to ETC of isolated mitochondria, regardless of any changes in gene expression. RET is mainly caused by excessive electron supply from Complex II and the blocked electron transfer pathway from Complex III to Complex IV. It is well known that RET produces ROS at the reaction site with ubiquinone in Complex I. [72]. It has been reported that inducing the appropriate range of mitochondrial ROS via an increase in RET by transgene of NDI1, a rotenone-insensitive alternative NADH dehydrogenase found in plants and fungi, which is able to effectively bypass Complex I and over-reduce ubiquinone, could extend the lifespan of *Drosophila* [216]. Interestingly, transgene of alternative oxidase (AOX), bypassing respiratory complexes III and IV by transferring electrons from reduced ubiquinone directly to O_2_, into skeletal muscle-specific COX15-deficient mice with severe myopathy, resulted in decreased ROS through avoiding RET [217]. Contrary to the expectation, these mice had a reduced lifespan and worsened myopathy, compared with the control COX15-deficient mice. Furthermore, the treatment of control COX15-deficient mice with NAC, which scavenged ROS such as RET-induced superoxide and subsequent H_2_O_2_, similarly reduced the lifespan. AOX introduction and supplementation of NAC caused the impaired AMPK/PGC-1α signaling by RET-induced ROS. Recently, it has been recognized again that mild inhibition of ETC is a target in the mechanism of action of several anti-diabetic drugs [192,218,219,220,221,222,223]. Thus, in certain situations RET can essentially be understood to be equivalent to the useful aspects of ROS in exercise. This may be one of the mechanisms of action of AX in improving mitochondrial energy metabolism. As noted above (Section 2.2.5), increases in ROS in physiological ranges impact the effects of exercise on the activation of AMPK [145]. In particular, the elevated production of superoxide and associated H_2_O_2_ at appropriate levels from “mitochondria” leads to the activation of AMPK, and extends lifespan in vivo, such as in *Drosophila*, *Caenorhabditis elegans* and mice [95,216,224,225]. These physiological responses against ROS can be considered as “***mitohormesis***” [226]. Again, AX does not interfere with the ameliorating effects of exercise on glucose metabolism and blood pressure, or the activation of AMPK by H_2_O_2_ [92]. There is an interesting report of the effect of AX on lifespan; it has been reported that AX extends the average lifespan of *C. elegans* wild-type and long-lived mutant *age-1* by about 16–30%, which codes an orthologue of mammal PI3K [227]. On the other hand, the *daf-16* mutant, an ortholog of the mammal Forkhead Box O1 (FoxO1) and FoxO3, did not achieve an extended lifespan in this study. FoxO family proteins are also known a target of Sirtuins, and the results of the AMPK/Sirtuins/PGC-1α pathway and the IGF-1 signaling pathway were confounded [228]. Further studies are required to explain the mechanism of action of AX. (see Section 2.2.5).

In association with these “*mitohormesis”*-like phenomena, in the last decade, very interesting investigations have been reported on the effects of other xanthophylls on mitochondria using genetic knockout models of carotenoid degrading enzymes BCDO2 [229,230]. Generally, carotenoids with strong provitamin A activity, such as β-carotene, are cleaved symmetrically by β, β−carotene-15,15′-oxygenase (BCDO1), localized in the cytoplasm, and the resulting metabolites are subsequently converted to retinoids. Unlike BCDO1, the substrates of BCDO2 are carotenoids, including xanthophylls and non-cyclic carotenes such as lycopene, and the C9 and C10 double-bond portions are cleaved asymmetrically. Since this enzyme is located in the mitochondria, BCDO2 knockout leads to accumulation of xanthophylls in mitochondria. Surprisingly, the administration of xanthophylls for BCDO2 knockout mice and cells developed severe steatosis and increased ROS production, instead of the expected antioxidant effects of xanthophylls [229]. To examine whether accumulation of xanthophylls affected mitochondrial activity, BCDO2 knockout mice were treated with the xanthophyll lutein, and then oxygen consumption was measured in respiratory State 3 (ADP-dependent) from Complex I, II, III, and IV. The oxygen consumption of each complex decreased in BCDO2^−/−^ mice fed a lutein diet, compared with the BCDO2^−/−^ mice fed a control diet. The addition of an uncoupler did not ameliorate this defect, indicating that lutein accumulation directly interfered with the electron transport chain. In addition, ADP/oxygen rate, a measure for the efficiency of oxidative phosphorylation, was not reduced. So, the mitochondria were structurally intact because the oxygen consumption and RCI in State 4 did not change, regardless of the existence of excessive lutein. Thus, abnormal accumulation of carotenoids generates ROS from ETC [229]. This is a very important discovery. The mechanism is not clear at this time, but it would be very interesting if the source of this ROS is due to xanthophyll-induced RET, or another similar mechanism. In contrast to rodents, human and monkey retinas and brains accumulate higher levels of xanthophylls than carotene, which may be due, in part, to the lower activity of human BCDO2 compared with mouse BCDO2 [231]. In addition, there are multiple isoforms of BCDO2 in humans, and their activity and localization are still under controversial. Therefore, it is possible that in humans there is an accumulation of xanthophylls at higher concentrations than rodents in the mitochondria.

In addition, under extreme conditions, such as when BCDO2 function was lacking, excessive carotenoid accumulation in the mitochondria also led to apoptosis [230]. This may be related to the apoptotic effect of AX on some cancer cells [232]. In another example, it has been shown that the metabolites of lycopene by BCDO2 prevent prostate cancer in in vivo transgenic mouse models [233,234,235]. In relation to AX, the effect of BCDO2-generated AX metabolites should be considered in the future, because it has only been studied in toxicological aspects, such as CYP induction in rats [125]. Perhaps independently of these features, BCDO2 itself probably functions as an anti-inflammatory factor through the modulation of several signaling pathways and gene expression [103,104,105,106,236,237].

These results may provide a better understanding of the many beneficial effects of AX and other carotenoids on energy metabolism and senescence that are mediated by the ROS-mediated activation of AMPK. This introduces a completely different aspect towards carotenoids than those previously considered. However, depending on the type of carotenoids and the BCDO2 activity of the individual, it may be related to cause of chronic inflammation and metabolic diseases, whereas the beneficial aspects of AX may only be revealed by its efficacy in skeletal muscle and its anti-inflammatory effects via its antioxidant activity in adipose tissue or liver. In conclusion, it is important to note that the BCDO2-mediated action needs to work in tandem with the protective antioxidant activity of carotenoids on biological membranes. Since it has been reported that the AX treatment of mitochondria isolated from vitamin E-deficient rats significantly protected the activity of the respiratory chain through the inhibition of mitochondrial lipid peroxidation by Fe^2+^ addition, it is likely that mitochondrial function itself is not impaired by AX, regardless of whether ROS is generated by AX or not [80]. It is conceivable from the literature presented in this review that AX is a very unique compound that prevents the structural destruction of proteins and lipids in mitochondria associated with highly reactive ROS-induced peroxidation reactions, such as hydroxyl radicals, lipid free radicals, and singlet oxygen, without affecting mitochondria-derived superoxide or H_2_O_2_ signaling. There is an interesting report that proves this concept: a comparison of mitochondrial function during heat stress using a skeletal muscle cell model between quercetin under heat stress [238]. Quercetin is a well-known polyphenolic compound which has antioxidant activity and promotes mitochondria biogenesis via the AMPK/PGC-1 pathway, as well as AX [239,240,241,242]. This study shows that only AX, not quercetin, was able to maintain mitochondrial membrane potential and mitochondria biogenesis, then keep its morphological and functional integrity under heat stress at 43 °C which generated higher levels of ROS from mitochondria in a skeletal muscular cell model. In this model, oxidative stress was evaluated with DHE and 2′,7′-dichlorodihydrofluorescein diacetate (H_2_DCFDA), a whole-cell ROS marker with relatively low specificity. According to the results, only AX, not quercetin, could suppress heat stress-induced ROS. Although it is difficult to separate the physical effects of AX on lipid peroxidation from the mitochondrial pathway via the AMPK/PGC-1α pathway, this result is an example of the cooperative functioning of both to maintain mitochondrial health. This effect was not observed with quercetin, indicating AX’s superiority in terms of maintaining mitochondrial integrity.

The discussions in this section are only circumstantial speculations based on the consistency of the current data. The authors hope that these new and exciting speculations will promote research in this area in the future.

## 3. Prospect of Astaxanthin for Human Health Promotion 

In rodents such as mice and rats, effective concentrations of AX were probably achieved at the doses used in the study in the targeted organs, and the medications were considered to be effective. Importantly, the doses of AX given to animals in the pharmacological studies presented in this review were quite high. The concentration of AX in the blood of humans and rodents deviates greatly, with the former reaching considerably higher concentrations than the latter [49,108,127,243,244,245]. In humans, although differences in absorption were observed in each clinical trial, this was thought to be due to dietary conditions, formulation, and individual differences. Therefore, it can be confidently expected that the benefits of AX for human subjects can be demonstrated by designing the formulation and administration method. Although they still remain to be improved, we summarized the human clinical studies reported to date on the antioxidant effects of AX (Table 1), as well as its impact on physical activity (Table 2) and cardiovascular, endocrine, and metabolic effects (Table 3). Based on the outcomes presented in Table 1, Table 2 and Table 3, AX can be expected to be especially useful in the prevention of metabolic diseases associated with obesity, T2DM, and sarcopenia, based on the mechanisms described in this review. The effects of AX are only mild, based on the results of clinical studies, and are additive to exercise, so it should be used in combination with standard therapeutic interventions and exercise therapy. Therefore, further research studies are warranted to elucidate the exact mechanism of action in more detail and consider the interaction with the mechanism of medication. The authors strongly expect that AX will contribute to improve people’s health.

## 4. Conclusions

In conclusion, AX not only acts on multiple biological defenses through the direct and indirect effects of its strong antioxidant activity, but also contributes to the maintenance and enhancement of mitochondrial activity by directly acting on the AMPK/Sirtuins/PGC-1α pathway, and other pathways. This action should be fully taken into account for a better understanding of the bioactivity of AX and other carotenoids. The activation of mitochondria and their function as biological defenses should be more effective with regard to the diverse diseases related to energy metabolism and aging (e.g., metabolic syndrome including T2DM, dyslipidemia and steatohepatitis, and frailty in aging, including cognitive impairment, cachexia and sarcopenia).

## Figures and Tables

**Table 1:** Human clinical studies with astaxanthin (AX) that examined oxidative stress markers.

Author/Year/Reference	Study Design	Subjects	Dose	Duration	Outcome
McAllister M.J. et al., 2021 [32]	Randomized, double-blind, placebo-controlled, crossover study	14 healthy subjects	0, 6 mg/day	4 weeks	Glutathione was ∼7% higher following AX compared with placebo (*p* < 0.05). No effect on plasma hydrogen peroxide or malondialdehyde (MDA; *p* > 0.05). Advanced oxidation protein products (AOPP) reduced by ∼28% (N.S.; *p* = 0.45).
Petyaev I.M., et al., 2018 [33]	Randomized, blinded, four-arm, prospective study	32 subjects with oxidative stress, 8 subjects taking AX only	0, 7 mg/day *	4 weeks	Reduced serum oxidized LDL by 55.4% after 4 weeks (*p* < 0.05). Reduced MDA by 52.7% after 4 weeks (*p* < 0.05).
Chalyk, N. et al., 2017 [34]	Open-label, prospective study	31 subjects; 18 obese, 8 overweight, 5 healthy weight	4 mg/day	92 days	Plasma MDA decreased with AX by 11.2% on day 15 and by 21.7% on day 29 (N.S.)
Hashimoto H. et al., 2016 [35]	Open-label, prospective study	35 subjects during cataract surgery	6 mg/day	2 weeks	Superoxide anion scavenging activity (U/mL) 18.2 ± 4.1 at 0 weeks reduced to 19.9 ± 3.6 after 2 weeks of supplementation compared with baseline, *p* < 0.05. Total hydroperoxides (U CARR) from 1.16 ± 0.18 at 0 weeks reduced to 1.04 ± 0.31 after 2 weeks of supplementation compared with baseline, *p* < 0.05
Baralic, I. et al., 2015 [36]	Randomized, double-blind, placebo-controlled, prospective study	40 healthy subjects (soccer players)	0, 4 mg/day	90 days	Improved prooxidant-antioxidant balance (PAB; *p* < 0.05)
Baralic I. et al., 2013 [37]	Randomized, double-blind, prospective study	40 healthy subjects (soccer players)	0, 4 mg/day	90 days	Protected thiol groups against oxidative modification (increase in -SH groups, *p* < 0.05; improved PON1 activity towards paraoxon and diazoxon, *p* < 0.05 and *p* < 0.01, respectively)
Hashimoto, H. et al., 2013 [38]	Open-label, prospective study	35 cataract patients	6 mg/day	2 weeks	Reduced total hydroperoxides (hydrogen peroxides, lipid peroxides, and peroxides of protein in aqueous humor; *p* < 0.05), increased superoxide scavenging activity (*p*< 0.05)
Choi H.D. et al., 2011 [39]	Randomized, two-arm, prospective study	23 obese and overweight subjects	5 and 20 mg/day	3 weeks	5 mg/day: MDA decreased by 34.6%, isoprostane (ISP) decreased by 64.9%, superoxide dismutase (SOD) increased by 193%, and total antioxidant capacity (TAC) increased by 121% after 3 weeks compared with baseline (*p* < 0.01). 20 mg/day: MDA decreased by 35.2%, ISP decreased by 64.7%, SOD increased by 194%, and TAC increased by 125% after 3 weeks compared with baseline (*p* < 0.01).
Choi, H.D. et al., 2011 [40]	Randomized, double-blind, placebo-controlled, prospective study	27 overweight subjects	0, 20 mg/day	12 weeks	MDA reduced by 17.3% and 29% after 8 and 12 weeks compared with placebo (*p* < 0.01), isoprostane (ISP) reduced by 40.2% and 52.9% after 8 and 12 weeks compared with placebo (*p* < 0.01), superoxide dismutase (SOD) increased by 124.8% after 12 weeks compared with placebo (*p* < 0.01), and total antioxidant capacity (TAC) increased by 130.1% after 12 weeks compared with placebo (*p* < 0.05) (See Table 3 for other outcomes.)
Hashimoto H. et al., 2011 [41]	Open-label, prospective study	35 cataract patients	6 mg/day	2 weeks	Reduced total hydroperoxides (hydrogen peroxides, lipid peroxides, and peroxides of protein in aqueous humor; *p* < 0.05)
Kim, J.H. et al., 2011 [42]	Randomized, Repeated, measured, prospective study	39 heavy smokers, 39 non-smokers	0, 5, 20, or 40 mg/day	3 weeks	5 mg/day: MDA and ISP significantly lower after 2 and 3 weeks compared with baseline in smokers (*p* < 0.05). SOD and TAC significant increase after 1, 2, and 3 weeks compared with baseline in smokers (*p* < 0.05) 20 mg/day: MDA and ISP significantly lower after 1, 2, and 3 weeks compared with baseline in smokers (*p* < 0.05). SOD and TAC significant increase after 1, 2, and 3 weeks compared with baseline in smokers (*p* < 0.05). 40 mg/day: MDA and ISP significantly lower after 1, 2, and 3 weeks compared with baseline in smokers (*p* < 0.05). SOD and TAC significant increase after 2 and 3 weeks compared with baseline in smokers (*p* < 0.05)
Nakagawa K. et al., 2011 [43]	Randomized, double-blind, placebo-controlled, prospective study	30 healthy subjects	0, 6, 12 mg/day	12 weeks	6 mg/day: reduction in total phospholipid hydroperoxides (PLOOH) after 12 weeks compared with baseline (*p* < 0.01) and compared with placebo (*p* < 0.05). Reduced phosphatidyl-ethanolamine hydroperoxide (PEOOH) after 12 weeks compared with baseline (*p* < 0.05) and compared with placebo (*p* < 0.05). 12 mg/day: 48% reduction in total PLOOH after 12 weeks compared with baseline (*p* < 0.01) and 35% less total PLOOH at 12 weeks compared with the control group (*p* < 0.05). The 12 mg/day group had 46% less phosphatidylcholine hydroperoxide (PCOOH) at 12 weeks compared with baseline (*p* < 0.01).
Peng L. et al., 2011 [44]	Randomized, placebo-controlled study	115 healthy subjects	0, 40 mg/day	90 days	Comparing with the control group, MDA contents in the test group decreased significantly (*p* < 0.01), and SOD and GSH-Px activities increased significantly (*p* < 0.01).
Park J.S. et al., 2010 [45]	Randomized, double-blind, placebo-controlled, prospective study	42 healthy subjects	2 or 8 mg/day	8 weeks	2 mg/day: Concentrations of plasma 8-hydroxy-2′-deoxyguanosine reduced after 4 weeks and 8 weeks compared with placebo (*p* < 0.05). 8 mg/day: Concentrations of plasma 8-hydroxy-2′-deoxyguanosine reduced after 4 weeks and 8 weeks compared with placebo (*p* < 0.05)
Iwabayashi M. et al., 2009 [46]	Open-label, prospective study	35 healthy subjects (with high oxidative stress)	12 mg/day	8 weeks	Increased blood biological antioxidant potential (BAP; +4.6%, *p* < 0.05)
Yamada T. et al., 2010 [47]	Open-label,prospective study	6 healthy subjects and 6 Sjoegren’s syndrome subjects	12 mg/day	2 weeks	Reduced protein oxidation (−10%, *p* < 0.05)
Fassett, R.G. et al., 2008 [48]	Randomized, double-blind, placebo-controlled, prospective study	58 renal transplant recipients	0, 12 mg/day	12 months	Total plasma F2-isoprostanes reduced by 23.0% in placebo and 29.7% in AX groups (N.S.)
Karppi, J. et al., 2007 [49]	Randomized, double-blind, placebo-controlled, prospective study	39 healthy subjects	0, 8 mg/day	3 months	Decreased oxidation of fatty acids in healthy men (*p* < 0.05)
Kim Y.K. et al., 2004 [50]	Open-label, prospective study	15 healthy postmenopausal women	0, 2, 8 mg/day	8 weeks	Decreased plasma TBARS levels: 2 mg group from 1.42 ± 0.18 to 1.13 ± 0.18 nM/mg (*p* < 0.05). 8 mg AX group from 1.62 ± 0.14 nM/mg to 1.13 ± 0.12 nM/mg after 8 weeks (*p* < 0.05). Increased TAS from 0.85 ± 0.42 mM/L to 1.90 ± 0.58 mM/L in the 8 mg group. Urinary 8-isoprostanes excretion did not decrease significantly. (See Table 3 for other outcomes.)

* In addition to AX, other nutrients such as antioxidants were used in the study.

**Table 2:** Human clinical studies of AX on physical performance, endurance and fatigue.

Author/Year/Reference	Study Design	Subjects	Dose	Duration	Outcome
<Subjects: healthy athletes, high daily physical activity>					
Brown, R.D. et al., 2021 [193]	Randomized, double-blind, placebo-controlled, crossover study	12 recreationally trained male cyclists 27.5 ± 5.7 years, VO_2peak_: 56.5 ± 5.5 mL⋅kg^−1^⋅min^−1^, W_max_: 346.8 ± 38.4 W	0, 12 mg/day	7 days	Completion time of the 40-km cycling time trial improved by 1.2 ± 1.7% with AX supplementation, from 70.76 ± 3.93 min in the placebo condition to 69.90 ± 3.78 min in the AX condition (mean improvement time = 51 ± 71 s, *p* = 0.029, g = 0.21). Whole body fat oxidation rate was also greater in the AX group between 39–40 km (+0.09 ± 0.13 g⋅min^−1^, *p* = 0.044, g = 0.52) and respiratory exchange ratio was lower (−0.03 ± 0.04, *p* = 0.024, g = 0.60).
Talbott I. et al., 2018 [194]	Randomized, double-blind, placebo-controlled, prospective study	28 recreational runners (42 ± 8 years)	0, 12 mg/day	8 weeks	Reduced average heart rate at submaximal endurance intensities (aerobic threshold, AeT and anaerobic threshold, AT), but not at higher “peak” intensities.
Klinkenberg L.J. et al., 2013 [195]	Randomized, double-blind, placebo-controlled prospective study	32 well-trained male cyclists 25 ± 5 years, V˙O_2_^peak^ = 60 ± 5 mL·kg^−1^·min^−1^, W_max_ = 5.4 ± 0.5 W·kg^−1^	0, 20 mg/day *	4 weeks	N.S; effect on exercise-induced cardiac troponin T release (*p* = 0.24), changes in antioxidant capacity markers (trolox equivalent antioxidant capacity, uric acid, and malondialdehyde). Markers of inflammation (high-sensitivity C-reactive protein) and exercise-induced skeletal muscle damage (creatine kinase).
Res T. et al., 2013 [196]	Randomized, double-blind, placebo-controlled, prospective study	32 trained male cyclists or triathletes 25 ± 1 years, V˙O_2_^peak^ = 60 ± 1 mL·kg^−1^·min^−1^, W_max_ = 395 ± 7 W	0, 20 mg/day	4 weeks	N.S; total plasma antioxidant capacity (*p* = 0.90) or attenuated malondialdehyde levels (*p* = 0.63). Whole-body fat oxidation rates during submaximal exercise (from 0.71 +/− 0.04 to 0.68 ± 0.03 g⋅min^−1^ and from 0.66 ± 0.04 to 0.61 ± 0.05 g⋅min^−1^ in the placebo and AX groups, respectively; *p* = 0.73), time trial performance (from 236 ± 9 to 239 ± 7 and from 238 ± 6 to 244 ± 6 W in the placebo and AX groups, respectively; *p* = 0.63).
Djordjevic B. et al., 2011 [197]	Randomized, Double-blind, placebo-controlled, prospective study	32 male elite soccer players	0, 4 mg/day	90 days	Changes in elevated O2-¯ concentrations after soccer exercise were statistically significant only in the placebo group (exercise × supplementation effect, *p* < 0.05); TAS values decreased significantly only in the placebo group after exercise (*p* < 0.01). After intervention, total SH group content increased (21% and 9%, respectively), and the effect of AX was marginally significant (*p* = 0.08). Basal SOD activity was significantly reduced in both the placebo and AX groups at the end of the study (main training effect, *p* < 0.01). Post-exercise CK and AST levels were significantly lower in the AX group than in the placebo group (*p* < 0.05)
Earnest C.P. et al., 2011 [198]	Randomized, double-blind, placebo-controlled, prospective study	14 amateur endurance-trained subjects 18–39 years, V˙O_2_^peak^ = 52.84 ± 3.5 mL·kg^−1^·min^−1^, W_max_ = 330 ± 26 W	0, 4 mg/day	28 days	Improved performance in the 20-km cycling time trial in the AX group (n = 7, −121 s; 95% CI, −185, −53), but not in the placebo group (n = 7, −19 s; 95% CI, −84, 45). AX group significantly increased power output (20 W; 95% CI, 1, 38), whereas the placebo group did not (1.6 W; 95% CI, −17, 20). N.S; carbohydrate, fat oxidation and blood indices indicative of fuel mobilization.
Bloomer, R.J. et al., 2005 [199]	Randomized, placebo-controlled, prospective study	20 resistance trained male subjects (25.1 ± 1.6 years)	0, 4 mg/day *	3 months	N.S; Muscle soreness, creatine kinase (CK), and muscle performance were measured before and through 96-h post-eccentric exercise
Sawaki K. et al., 2002 [200]	Randomized double-blind placebo-controlled, prospective study	16 healthy adult male subjects	0, 6 mg/day	4 weeks	In the AX group, the serum lactate concentration after 2 min of activity (1200 m run) was significantly lower than that in the control group.
<Subjects: healthy subjects>					
Kawamura A. et al., 2021 [201]	Randomized controlled open-label, prospective study	26 healthy male subjects	N/A (1 mg AX/100 g salmon) *	10 weeks	The skeletal muscle mass was higher after training than before training in both control and intervention groups (*p* < 0.05). Increased maximal voluntary contraction after training in the intervention group (*p* < 0.05), but not significantly increased in the control group. (See Table 3 for other outcomes.)
Fleischmann C. et al., 2019 [202]	Randomized, double-blind, placebo-controlled, prospective study	22 healthy subjects	0, 12 mg/day	30 days	Decreased raise in blood lactate caused by the VO_2 Max_ test; AX group (9.4 ± 3.1 and 13.0 ± 3.1 mmole⋅L^−1^ in the AX and placebo groups, respectively *p* < 0.02). Change in oxygen uptake during recovery (−2.02 ± 0.64 and 0.83 ± 0.79% of VO2 _Max_ in the AX and placebo group, respectively, *p* = 0.001). N.S; anaerobic threshold or VO2 Max. physiological or biochemical differences in the heat tolerance test (HTT) (2 h walk at 40 °C, 40% relative humidity.
Takami M. et al., 2019 [203]	Open-label, prospective study	20 healthy young male subjects	c.a, 4.5 mg/day * from salmon	4 weeks	Increased maximum workload by training in both groups (*p* = 0.009), and increased oxygen consumption during exercise in the antioxidant group only (*p* = 0.014). There were positive correlations between maximum workload and fat (*r* = 0.575, *p* = 0.042) and carbohydrate oxidations (r = 0.520, *p* = 0.059) in the antioxidant group. (See Table 3 for other outcomes.)
Imai A. et al., 2018 [204]	Randomized, double-blind, placebo-controlled, crossover study	42 healthy subjects	0, 6 mg/day *	4 weeks	Elevated PCOOH levels during mental and physical tasks were attenuated by AX supplementation. Improved recovery from mental fatigue compared with the placebo. No differences were found between AX and the placebo in other secondary outcomes, such as subjective feelings, work efficiency, and autonomic activity.
Hongo N. et al., 2017 [205]	Randomized, double-blind placebo-controlled, prospective study	39 healthy subjects	0, 12 mg/day *	12 weeks	Intent-to-treat (ITT) analysis; fatigue after physical and mental stress was significantly lower in the AX group than in the placebo at week 8; the change in POMS Friendliness was significantly higher in the AX group than in the control group at week 8; the rate of change in BAP values at week 12 was not significantly different between the AX and control groups. The rate of change in BAP values at week 12 was not significantly different between the AX group and the control.
Malmstena C.L.L. et al., 2008 [206]	Randomized, double-blind, placebo-controlled, prospective study	40 young healthy subjects (17–19 years)	0, 4 mg/day	3 months	Increased average number of knee bending (squats) increased by 27.05 (from 49.32 to 76.37, AX group) vs. 9.0 (from 46.06 to 55.06, placebo subjects), *p* = 0.047.
Tajima T. et al., 2004 [207]	Randomized, double-blind, placebo-controlled, crossover study	18 healthy subjects (35.7 ± 4 years)	0, 5 mg/day	2 weeks	Increased in CV_RR_ and HF/TF (Heart rate variability) were significant during exercise at 70% maximum heart rate (HRmax) intensity (*p* < 0.05). Also, after the AX supplementation, decreased minute ventilation (V_E_) during exercise at 70% HRmax (*p* < 0.05). Decreased LDL cholesterol (chol) (*p* < 0.05) and respiratory quotient after exercise.
<Subjects: elderly subjects>					
Liu S.Z. et al., 2021 [189]	Randomized, double-blind, placebo-controlled, prospective study	42 elderly subjects (65–82 years)	0, 12 mg/day *	12 weeks	In endurance training (ET), specific muscular endurance was improved only in the AX group (Pre 353 ± 26 vs. Post 472 ± 41) and submaximal graded exercise test duration was improved in both groups (placebo 40.8 ± 9.1% vs. AX 41.1 ± 6.3%). The increase in fat oxidation at low intensity after ET was greater in AX (placebo 0. 23 ± 0.15 g vs. AX 0.76 ± 0.18 g), and was associated with reduced carbohydrate oxidation and improved exercise efficiency in men, but not in women.
Liu S.Z. et al., 2018 [190]	Randomized double-blind, placebo-controlled, prospective study	42 elderly subjects (65–82 years)	0, 12 mg/day *	12 weeks	Administration of AX increased maximal voluntary force (MVC) by 14.4% (± 6.2%, *p* < 0.02), tibialis anterior muscle size (cross-sectional area, CSA) by 2.7% (± 1.0%, *p* < 0.01), and specific impulse increased by 11.6% (MVC/CSA, ± 6.0%, *p* = 0.05), respectively, whereas placebo treatment did not alter these characteristics (MVC, 2.9% ± 5.6%; CSA, 0.6% ± 1.2%; MVC/CSA, 2.4 ± 5.7%; all *p* > 0.6).
Fujino H. et al., 2016 [191]	Randomized, double-blind, placebo-controlled, prospective study	29 community-dwelling healthy elderly subjects (80.9 ± 1.5 years.)	0, 12 mg/twice a day *	3 months	Decrease in d-ROM values with AX group (*p* < 0.01), but not the placebo group; the AX group had a therapeutic effect on 6-min walking distance compared with the placebo group (*p* < 0.05). AX group had an increase in distance and number of steps in the 6-min walking test compared with the placebo group. Furthermore, the rate of increase in blood lactate levels after walking was lower in the AX group than in the placebo group (*p* < 0.01).

* In addition to AX, other nutrients such as antioxidants were used in the study.

**Table 3:** Human clinical studies of AX on endocrinology, cardiovascular and metabolism.

Author/Year/Reference	Study Design	Subjects	Dose	Duration	Outcome
Shokri-Mashhadi, N. et al., 2021 [208]	Randomized, double-blind, placebo-controlled, prospective study	44 patients with type 2 diabetes	0, 8 mg/day	8 weeks	Decrease plasma levels of MDA and IL-6 (*p* < 0.05) and decrease the expression level of miR-146a, associated with inflammatory markers (fold change: −1/388) (*p* < 0.05).
Kawamura A. et al., 2021 [201]	Randomized controlled Open-label, prospective study	26 healthy male subjects	*N/A* (1 mg AX/100 g salmon) *	10 weeks	Higher resting oxygen consumption after training in the intervention group only (*p* < 0.05). Serum carbonylated protein level as an oxidative stress marker tended to be lower immediately after exercise than before exercise in the intervention group only (*p* = 0.056). (See Table 2. for other outcomes.)
Kato T. et al., 2020 [209]	Open-label, prospective study	16 patients with systolic heart failure	12 mg/day *	3 months	Increased left ventricular ejection fraction (LVEF) from 34.1 ± 8.6% to 38.0 ± 10.0% (*p* = 0.031) and 6-min walk distance increased from 393.4 ± 95.9 m to 432.8 ± 93.3 m (*p* = 0.023). Significant relationships were observed between percent changes in dROM level and those in LVEF.
Chan K. et al., 2019 [210]	Randomized controlled Open-label, prospective study	54 patients with type 2 diabetes	0, 6, 12 mg/day	8 weeks	Increased plasma AX levels and decreased fasting plasma glucose and HbA1c levels. In 12 mg AX group, reduced in plasma triglyceride, total chol and LDL levels. Lowered changes in plasma IL-6 and TNF-α levels and plasma vWF level and higher changes in AT-III level. In 12 mg AX group, decreased changes in plasma FVII and PAI-1 levels.
Takami M. et al., 2019 [211]	Open-label, prospective study	20 healthy young male subjects	c.a, 4.5 mg/day * from salmon	4 weeks	Higher carbohydrate oxidation during rest in the post-training than that in the pre-training only in the antioxidant group. More decreased levels of serum insulin and HOMA-IR after training were observed in the antioxidant group than in the control group. (See Table 2. for other outcomes.)
Mashhadi N.S. et al., 2018 [163]	Randomized, double-blind, placebo-controlled, prospective study	44 participants with type 2 diabetes	0, 8 mg/day	8 weeks	Increased the serum adiponectin concentration, reduced visceral body fat mass (*p* < 0.01), serum triglyceride and VLDL chol concentrations, systolic blood pressure, fructosamine concentration (*p* < 0.05) and marginally reduced the plasma glucose concentration (*p* = 0.057).
Canas J. A. et al., 2017 [211]	Randomized, double-blind, placebo-controlled, prospective study	20 children with simple obesity (BMI > 90%)	500 μg/day * (MCS)	6 months	Mixed-carotenoid supplementation (MCS) increased β-carotene, total adiponectin, and high-molecular-weight adiponectin in plasma compared with placebo; MCS decreased BMI z-score, waist-to-height ratio, and subcutaneous adipose tissue compared with placebo. AX was used as a part of MCS.
Takemoto M. et al., 2015 [212]	Case report	1 Werner syndrome patient	12 mg/day *	6 months	Improved blood transaminase concentrations before AX intervention and 3 and 6 months after initiation were: AST 40 IU/L, 41 IU/L, and 20 IU/L; ALT 69 IU/L, 62 IU/L, and 34 IU/L; GGT 38 IU/L, 41 IU/L, and 35 IU/L; and cholinesterase 360 IU/L, 366 IU/L, and 331 IU/L, respectively. Liver-to-spleen Hounsfield units on CT were 0.41 before AX initiation, 0.71 at 3 months, and 0.94 at 6 months. No significant changes after AX intervention in hyaluronic acid, a marker of liver fibrosis; high-sensitivity C-reactive protein, a marker of inflammation; and MDA-modified LDL.
Ni Y. et al., 2015[108]	Randomized, single-blind, placebo-controlled, prospective study	12 NASH patients	12 mg/day	24 weeks	Improved steatosis (*p* < 0.05), marginally improved lobular inflammation (*p* = 0.15) and NAFLD activity score (*p* = 0.08)
Choi H.D. et al., 2011 [40]	Randomized, double-blind, placebo-controlled, prospective study	27 overweight subjects (BMI >25.0 kg/m^2^)	0, 20 mg/day	12 weeks	Decreased LDL chol and ApoB. (See Table 1. For other outcomes.)
Yoshida H. et al., 2010 [161]	Randomized, ouble-blind, placebo-controlled, prospective study	61 non-obese subjects with fasting serum triglyceride of 120–200 mg/dL and without diabetes and hypertension	0, 6, 12, 18 mg/day	12 weeks	Multiple comparison: triglycerides were significantly decreased by 12 and 18 mg/day and HDL-cholesterol was significantly increased by 6 and 12 mg. Serum adiponectin was increased by AX (12 and 18 mg/day), and changes in adiponectin were positively correlated with changes in HDL-chol.
Satoh A. et al., 2009 [213]	Open-label, prospective study	20 subjects at risk for developing metabolic syndrome (from 127 healthy subjects)	4, (8, 20) mg/day	4 weeks.	When subjects who met the diagnostic criteria for metabolic syndrome in Japan (SBP ≥ 130 mmHg, DBP ≥ 85 mmHg, TG ≥ 150 mg/dL, FG ≥ 100 mg/dL) at the start of the study were selected from 4 mg group, significant decreased in SBP(*p* < 0.01). On the other hand, there was no significant decrease in DBP. Reduced TG after treatment (218 mg/dL) than the baseline value (292 mg/dL), marginally reduced fasting glucose after the intervention (*p* < 0.1).
Uchiyama A. et al., 2008 [162]	Open-label, prospective study	17 subjects at risk for developing metabolic syndrome	8 mg twice day	3 months	Significant decreases plasma HbAlc (*p* = 0.0433) and TNF-α levels (*p* = 0.0022) and increase adiponectin concentration (*p* = 0.0053). N.S: body weight, BMI and waist circumference.
Fukamauchi M. et al., 2007 [214]	Randomized, double-blind, placebo-controlled, prospective study	32 healthy subjects	0, 6 mg/day	6 weeks	Synergistic effects of AX intake (12 mg/day, 6 weeks) and aerobic exercise (walking) were studied. AX contributed to reduction of body fat and suppressed the increase in blood lactate level after exercise.
Kim Y.K. et al., 2004 [50]	Open-label, prospective study	15 healthy postmenopausal female subjects	0, 2, 8 mg/day	8 weeks	Increase HDL-chol levels in 2 mg and 8 mg group increased significantly after 8 weeks from 50.6 ± 5.8 to 60.4 ± 7.1 mg/dL, 44.4 ± 10.7 to 49.4 ± 2.7 mg/dL respectively (*p* < 0.05). In the 2 mg group, triglyceride decreased significantly from 171.6 ± 67.4 mg/dL to 145.8 ± 5.1 mg/dL (*p* < 0.05). (See Table 1. For other outcomes.)

* In addition to AX, other nutrients such as antioxidants were used in the study.

## Data Availability

All data underlying the results are available as part of the article and no additional source data are required.

## Supplementary Materials

The following are available online at https://www.mdpi.com/article/10.3390/nu14010107/s1, Figure S1: AX elevated NAD^+^ levels in C2C12 myoblasts. Figure S2: AX prevented age related glucose intolerance and insulin resistance in male C57BL/6J mice fed a normal diet (NC). Figure S3: Effect of AX on respiratory activity of isolated mitochondria from mouse liver.
