# Supplementary material for: Astaxanthin as a Novel Mitochondrial Regulator: A New Aspect of Carotenoids, beyond Antioxidants
Source: Nutrients. 2021 Dec 27;14(1):107. doi: 10.3390/nu14010107 (PMC8746862; doi:10.3390/nu14010107)
Supplement: Supplementary file 1 [file nutrients-14-00107-s001.zip › nutrients-1497237-supplementary.pdf]

Supplementary Material

Materials and Methods

Regents

Cell culture reagents were purchased from Invitrogen (Carlsbad, CA); astaxanthin (AX) and All other reagents were purchased from Sigma-Aldrich (St. Louis) and Wako Pure Chemicals (Japan).

Cell culture

C2C12 cells (American Type Culture Collection; ATCC® CRL-1772™, Manassas, Virginia) were cultured as described previously [1]. Briefly, C2C12 cells were grown to confluency (60–70%) in growth medium containing low glucose Dulbecco's Modified Eagle's Medium (DMEM, Thermo Fisher Scientific) with 10% fetal bovine serum (Gibco™ 10437-028) without antibiotics at 37° C in 5% CO_2, and then cells were treated with AX (50μM) and vehicle (0.2% dimethyl sulfoxide) for specified time. After treatment for 24 hours, cells were washed with phosphate-buffered. Metabolites were extracted from cells with water/methanol/chloroform (25:25:50 by volume). After centrifugation, the aqueous phase was isolated and dried using a SpeedVac SPD1010 (Thermo). For LC-MS analysis, the dried sample was reconstituted with 60 μl LC-MS grade water (Wako) and filtered through a 0.45 μm Millex filter unit (Millipore) [2].

Metabolomic Analysis by LC-MS

NAD^+ levels were determined by multiple reaction monitoring (MRM) mode using an Agilent 6460 Triple Quad mass spectrometer coupled to an Agilent 1290 HPLC system. Chromatographic conditions were used as previously described [2]. Data were given as a ratio to the mean MS count of the control group.

Animals

Five-week-old male C57BL/6J mice were purchased from Sankyo Laboratory Service (Tokyo, Japan). All animals were housed in a 12-h light/12-h dark cycle and allowed free access to food and water. The regular diet (normal chow [NC]; D12450B) and the AX pre-mixed diets (final AX content using commercially available AX powder was 0.02%) were purchased from Research Diets Inc. (New Brunswick, NJ)[1]. One week after habitation, from 6 week old, they were started fed an each diet. For *ex vivo* assay of mitochondria oxygen consumption, mice were sacrificed after anesthesia intraperitoneal injection at this timing. For metabolic challenges, mice in each treatment group received to either an intraperitoneal glucose tolerance test or an insulin tolerance test every 8 weeks for up to 48 weeks. After the test, the animals were kept at rest for two weeks and the another test were performed. After all tests were done, the mice were sacrificed by intraperitoneal injection with anesthesia. The animal care policies and procedures for the experiments were approved by the animal experiment committee at the University of Toyama.

Materials and Methods

Glucose tolerance test and insulin tolerance test

For the intraperitoneal glucose tolerance test, the mice were fasted for 18 h and were administered an intraperitoneal injection of glucose; 1 mg/g body weight (BW). For the intraperitoneal insulin tolerance test, mice fasted for 2–3 h and were administered an intraperitoneal injection of human insulin (0.8 units/kg BW) for the mice fed Blood samples were then collected from the tail vein at 0, (15), 30, 45, 60, 90, and 120 min after the injection for glucose/insulin measurement. The blood glucose levels obtained from the tail tip of the mice were measured using STAT STRIP Express 900 (Nova Biomedical, Waltham MA).

Mitochondrial Oxygen Consumption

Mouse liver mitochondria were isolated using a standard differential centrifugation protocol in 250 mM sucrose, 20 mM Tris-HCl, and 1 mM EGTA, pH 7.4 **[3]**. Mitochondrial oxygen consumption rate was measured on freshly prepared mitochondria at 37° C using a Clark-type O_2 electrode (Oxytherm System; Hansatech Instruments, Norfolk, U.K.) in a 1 mL chamber filled with MiR06 buffer, which is composed of MiR05 buffer and 280 U/mL catalase and using 1 mg mitochondrial proteins/mL **[4]**. AX was dissolved in dimethyl sulfoxide (DMSO) to 5 mM and this was used as the stock solution. This solution was added to the isolated mitochondria to a final concentration of 5 μM, and the same volume of DMSO was added to the Vechicle. Measurements were performed in the presence of either glutamate (10 mM)/malate (5 mM) (GM) or succinate (5 mM) with/without 3 μM rotenone as substrates after the addition of 300 μM ADP (State 3) followed by 2 μg/mL oligomycin (State 4). All analyses were repeated at least triplicate.

Supplementary Figure S1.

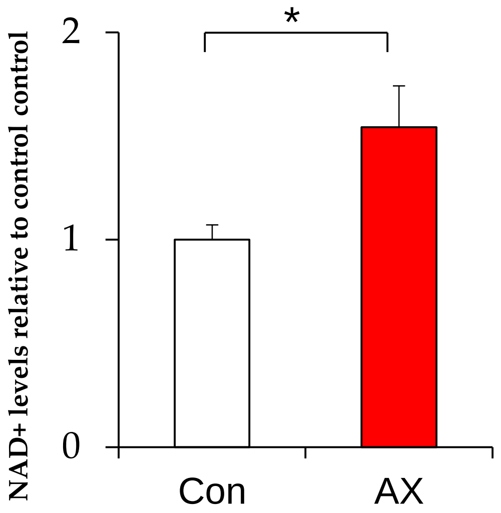

Supplementary Figure 1. AX elevated NAD^+ levels in C2C12 myoblast.

NAD^+ levels in C2C12 cells treated for 24 hr with 50 µM AX (n = 3). All values are presented as the means ± S.E.M. *p < 0.05, (Vehicle vs. AX). Statistical tests were performed as follows: Student's t test.

Supplementary Figure S2.

(A) i.p.GTT

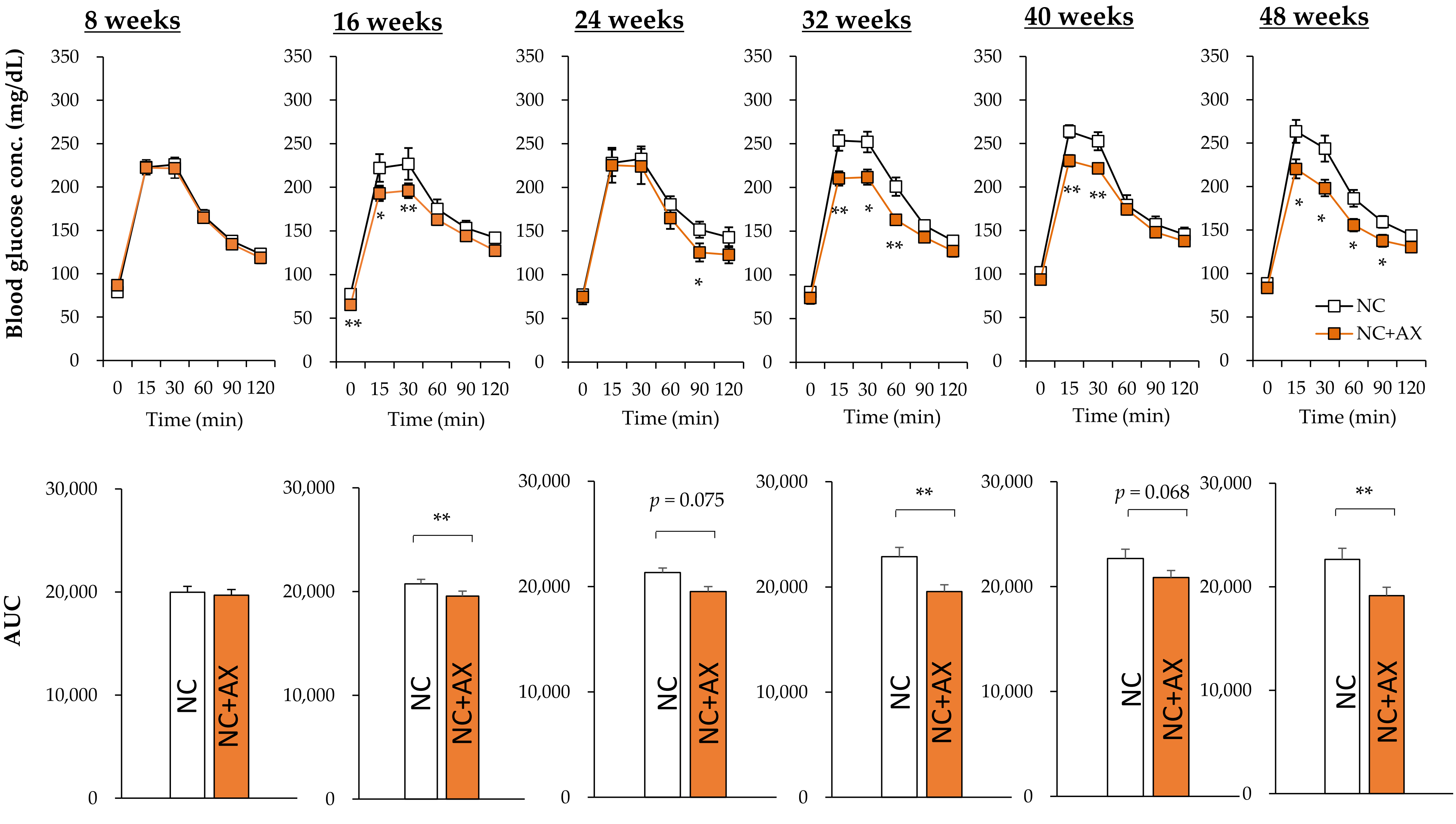

Supplementary Figure S2.

(B) ITT

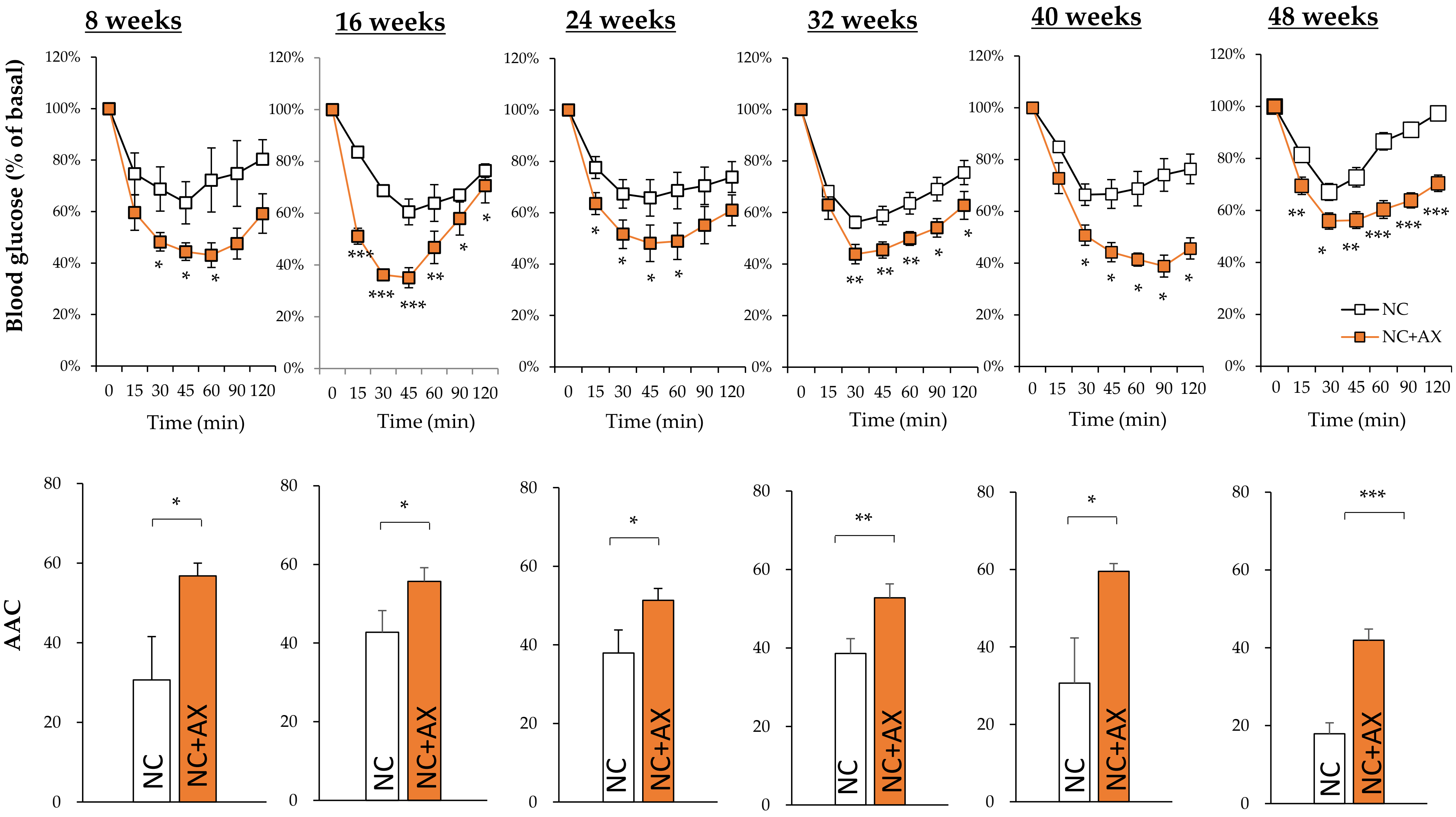

Supplementary Figure S2. AX prevented age-related glucose intolerance and insulin resistance in male C57BL/6J mice fed a normal diet (NC).

(A) Intraperitoneal glucose tolerance test (i.p.GTT) and (B) Intraperitoneal insulin tolerance test (IP-ITT) in the AX-treated NCmice compared with control NC mice for 8, 16, 24, 32, 40 and 48 weeks (n = 3–10 per group). The area under the curve (AUC) for GTT and the area above the curve (AAC) for ITT were also noted, respectively. Mice were started on each treatment at 6 weeks of age. All values are represented as means ± S.E.M. *$p < 0.05$, . **$p < 0.01$, . ***$p < 0.001$ (NC vs. NC+AX). Statistical tests were performed as follows: Student's *t*-test.

Supplementary Figure S3.

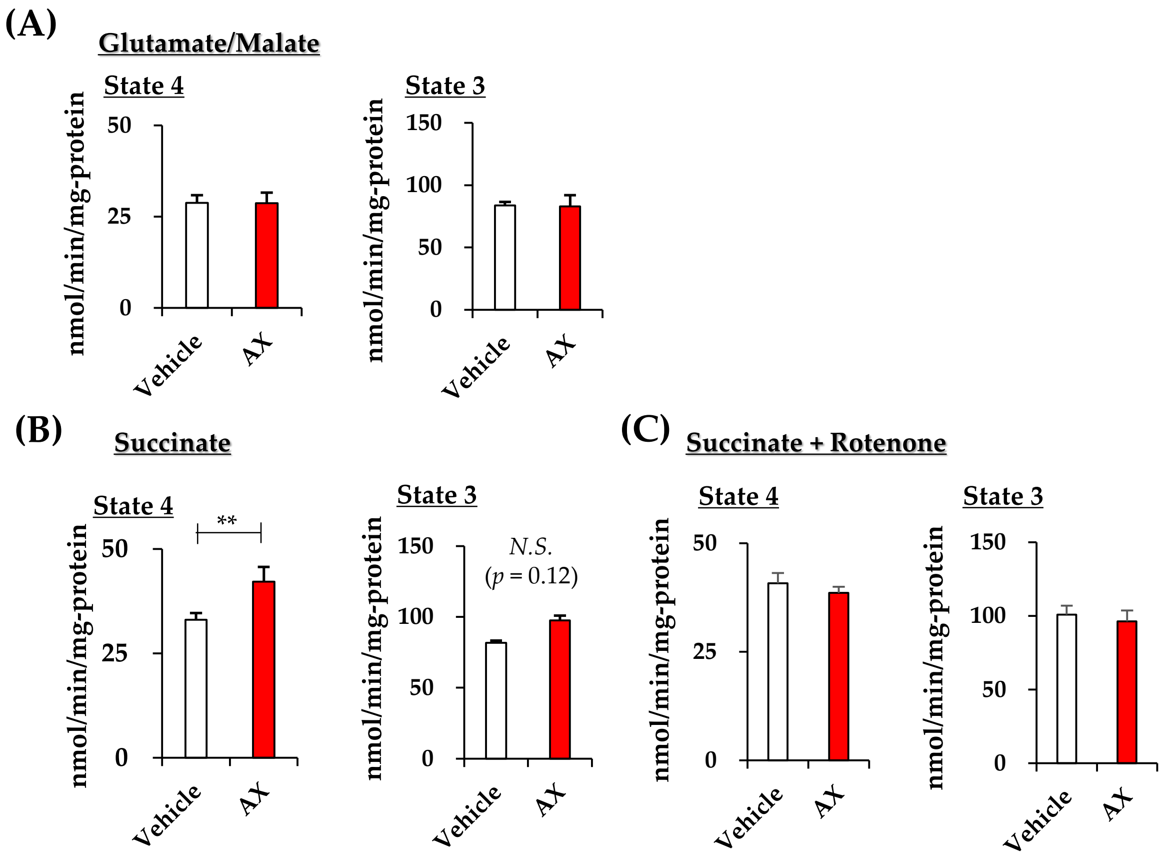

Supplementary Figure 3. Effect of AX on respiratory activity of isolated mitochondria from mouse liver.

Oxygen consumption rate of mouse liver mitochondria when the substrate is glutamate + malate (A) and succinate (B). Furthermore, when the substrate was succinate, rotenone, an inhibitor of Complex I, was added (C). All values are presented as the means ± S.E.M. (n = 7-12 in each treatment) **$p < 0.01$, (Vehicle vs. AX). Statistical tests were performed as follows: Student's t test.

References

1. Nishida, Y.; Nawaz, A.; Kado, T.; Takikawa, A.; Igarashi, Y.; Onogi, Y.; Wada, T.; Sasaoka, T.; Yamamoto, S.; Sasahara, M.; et al. Astaxanthin stimulates mitochondrial biogenesis in insulin resistant muscle via activation of AMPK pathway. Journal of cachexia, sarcopenia and muscle **2020**, *11*, 241-258, doi:10.1002/jcsm.12530.
2. Okabe, K.; Nawaz, A.; Nishida, Y.; Yaku, K.; Usui, I.; Tobe, K.; Nakagawa, T. NAD+ Metabolism Regulates Preadipocyte Differentiation by Enhancing alpha-Ketoglutarate-Mediated Histone H3K9 Demethylation at the PPARgamma Promoter. Front Cell Dev Biol **2020**, *8*, 586179, doi:10.3389/fcell.2020.586179.
3. Frezza, C.; Cipolat, S.; Scorrano, L. Organelle isolation: functional mitochondria from mouse liver, muscle and cultured filroblasts. *Nat. Protocols* **2007**, *2*, 287-295.
4. Fasching M, F.-A.M., Gnaiger E. Mitochondrial respiration medium - MiR06. *Mitochondr Physiol Network* **2016**, *14*, 1-4.
